# Supplementary material for: Systematic review and meta-analysis of pregnancy outcomes in women with polycystic ovary syndrome
Source: Nat Commun. 2024 Jul 4;15:5591. doi: 10.1038/s41467-024-49749-1 (PMC11224312; doi:10.1038/s41467-024-49749-1)
Supplement: Supplementary file 1 — Supplementary Information [file 41467_2024_49749_MOESM1_ESM.pdf]

Systematic Review and Meta-Analysis of Pregnancy Outcomes in women with Polycystic Ovary Syndrome

Supplementary Table 1. Characteristics of included studies

Study	Country	Design	Quality	PCOS	Controls	Matched characteristics	Outcomes
Diamant et al (1982)	Israel	Prospective	Poor	N: 70<br>Age*: 31<br>BMI†: NR	N: 2071<br>Age: 30<br>BMI: NR	-	PE<br>Instrumental<br>CS
Levran et al (1990)	Israel	Prospective	Poor	N: 76<br>Age: 27<br>BMI: NR	N: 95<br>Age: 28<br>BMI: NR	Age,<br>Weight	GDM
Wortsman et al (1991)	USA	Retrospective cohort	Poor	N: 53<br>Age: 29<br>BMI: NR	N: 2036<br>Age: NR<br>BMI: NR	-	GDM
Urman et al (1992)	Canada	Prospective	Moderate	N: 4<br>Age: NR<br>BMI: NR	N: 10<br>Age: NR<br>BMI: NR	Age	Miscarriage
Homburg et al (1993)	Israel	Prospective	Poor	N: 47<br>Age: NR<br>BMI: NR	N: 38<br>Age: NR<br>BMI: NR	-	Miscarriage
Lesser et al (1997)	USA	Retrospective cohort	Poor	N: 24<br>Age: 30<br>BMI: 28	N: 44<br>Age: 32<br>BMI: 23	-	GDM<br>GWG
Urman et al (1997)	Turkey	Retrospective cohort	Moderate	N: 47<br>Age: 28<br>BMI: 25	N: 100<br>Age: 28<br>BMI: 23	-	GDM<br>GH<br>PE<br>GWG
Fridstrom et al (1999)	Sweden	Retrospective cohort	Poor	N: 9<br>Age: 32<br>BMI: 25	N: 10<br>Age: 33<br>BMI: 23	Age	GDM<br>GH<br>PE<br>GWG<br>CS
Kashyap et al (2000)	Canada	Retrospective cohort	Poor	N: 22<br>Age: NR<br>BMI: NR	N: 27<br>Age: NR<br>BMI: NR	-	GH
Vollenhoven et al (2000)	Australia	Retrospective cohort	Moderate	N: 60<br>Age: NR<br>BMI: 27	N: 60<br>Age: NR<br>BMI: 27	Age<br>BMI<br>Ethnicity	GDM<br>Instrumental
Mikola et al (2001)	Finland	Retrospective cohort	Poor	N: 99<br>Age: 30<br>BMI: 26	N: 737<br>Age: 29<br>BMI: 23	-	GDM<br>GH<br>PE<br>CS
Wang et al (2001)	Australia	Prospective	Poor	N: 373<br>Age: 31<br>BMI: 26	N: 645<br>Age: 33<br>BMI: 24	-	Miscarriage
Bjercke et al (2002)	Norway	Retrospective cohort	Poor	N: 52<br>Age: 31<br>BMI: 26	N: 355<br>Age: 33<br>BMI: 22	-	GDM<br>GH<br>PE<br>Instrumental<br>CS
Sir-Petermann et al (2002)	Chile	Prospective	Moderate	N: 20<br>Age: NR<br>BMI: NR	N: 26<br>Age: NR<br>BMI: NR	Age	GDM
Haakova et al (2003)	Czech Republic	Retrospective cohort	High	N: 66<br>Age: 29<br>BMI: 24	N: 66<br>Age: 30<br>BMI: 23	Age<br>Weight	GDM<br>GH<br>GWG<br>CS
Turhan et al (2003)	Turkey	Retrospective cohort	Poor	N: 38<br>Age: 28<br>BMI: 32	N: 136<br>Age: 27<br>BMI: 24	-	GDM<br>GH<br>PE<br>GWG<br>IoL

							CS
Glueck et al (2004)	USA	Prospective	Poor	N: 119<br>Age: 31<br>BMI: 34	N: 251<br>Age: 29<br>BMI: 26	-	GDM<br>PE
Glueck et al (2004)	USA	Prospective	Poor	N: 95<br>Age: 33<br>BMI: 34	N: 251<br>Age: 29<br>BMI: 26	-	GDM<br>PE
Weerakiet et al (2004)	Thiland	Retrospective cohort	Moderate	N: 36<br>Age: 32<br>BMI: 24	N: 100<br>Age: 31<br>BMI: 22	Age	GDM<br>GH<br>PE<br>GWG<br>CS
Sir-Petermann et al (2005)	Chile	Prospective	Poor	N: 47<br>Age: 25<br>BMI: 28	N: 180<br>Age: 26<br>BMI: 26	Age<br>BMI<br>SES	GDM<br>GWG
Al-Ojaimi et al (2006)	Bahrain	Prospective	Poor	N: 134<br>Age: 29<br>BMI: 31	N: 479<br>Age: 28<br>BMI: 29	-	GDM<br>GH<br>PE<br>GWG
Dokras et al (2006)	USA	Retrospective cohort	High	N: 46<br>Age: NR<br>BMI: NR	N: 108<br>Age: NR<br>BMI: NR	Weight	Miscarriage<br>GDM<br>CS
Kovo et al (2006)	Israel	Retrospective cohort	Moderate	N: 33<br>Age: 30<br>BMI: 28	N: 66<br>Age: 31<br>BMI: 25	Age	GDM<br>GH<br>CS
Hu et al (2007)	UK	Prospective	High	N: 22<br>Age: 32<br>BMI: 24	N: 22<br>Age: 32<br>BMI: 24	Age<br>BMI<br>Ethnicity<br>Parity	GH<br>PE<br>EC
Palep-Singh et al (2007)	UK	Retrospective cohort	Poor	N: 120<br>Age: NR<br>BMI: NR	N: 95<br>Age: NR<br>BMI: NR	-	Miscarriage
Sir-Petermann et al (2007	Chile	Prospective	High	N: 48<br>Age: NR<br>BMI: NR	N: 51<br>Age: NR<br>BMI: NR	Age<br>SES	GDM<br>GH
Beydoun et al (2009)	USA	Retrospective cohort	Poor	N: 28<br>Age: 32<br>BMI: 31	N: 23<br>Age: 32<br>BMI: 24	-	Miscarriage
Bolton et al (2009)	Ireland	Retrospective cohort	Poor	N: 66<br>Age: 32<br>BMI: NR	N: 66<br>Age: 32<br>BMI: NR	Age<br>Parity	GDM
Gupta et al (2009)	India	Prospective	Moderate	N: 56<br>Age: NR<br>BMI: NR	N: 56<br>Age: NR<br>BMI: NR	Age<br>Weight	GDM<br>GH
Maliqueo et al (2009)	Chile	Prospective	Moderate	N: 30<br>Age: 27<br>BMI: 29	N: 34<br>Age: 27<br>BMI: 24	SES	GWG
Anderson et al (2010)	USA	Prospective	Poor	N: 39<br>Age: 30<br>BMI: 31	N: 31<br>Age: 32<br>BMI: 25	-	GWG<br>CS
Falbo et al (2010)	Italy	Prospective	High	N: 45<br>Age: 28<br>BMI: 25	N: 42<br>Age: 28<br>BMI: 25	Age<br>BMI	GH<br>PE
Li et al (2010)	China	Retrospective cohort	Poor	N: 34<br>Age: 32<br>BMI: NR	N: 70<br>Age: 32<br>BMI: NR	-	PE
Palomba et al (2010)	Italy	Prospective	Poor	N: 93<br>Age: NR<br>BMI: NR	N: 69<br>Age: NR<br>BMI: NR	-	Miscarriage<br>GDM<br>GH<br>PE
Palomba et al (2010)	Italy	Prospective	High	N: 70<br>Age: NR<br>BMI: NR	N: 69<br>Age: NR<br>BMI: NR	Age<br>BMI	Miscarriage<br>GDM<br>GH<br>PE<br>Instrumental<br>CS

De Leo et al (2011)	Italy	Prospective	Poor	N: 98<br>Age: 32<br>BMI: 28	N: 110<br>Age: 33<br>BMI: 27	-	Miscarriage<br>GDM<br>GH<br>PE
Dmitrovic et al (2011)	USA	Retrospective cohort	Poor	N: 17<br>Age: 29<br>BMI: 32	N: 17<br>Age: 31<br>BMI: 26	-	GDM
Nejad et al (2011)	Iran	Prospective	Moderate	N: 52<br>Age: NR<br>BMI: NR	N: 47<br>Age: NR<br>BMI: NR	-	Miscarriage
Nouh et al (2011)	Egypt	Prospective	High	N: 40<br>Age: NR<br>BMI: NR	N: 40<br>Age: NR<br>BMI: NR	Age<br>BMI	Miscarriage<br>GDM<br>GH<br>PE<br>CS
Mehrabian et al (2012)	Iran	Retrospective cohort	High	N: 40<br>Age: 27<br>BMI: 26	N: 40<br>Age: 28<br>BMI: 26	Age,<br>BMI<br>SES	GWG
Palomba et al (2012)	Italy	Prospective	High	N: 42<br>Age: 28<br>BMI: 28	N: 84<br>Age: 28<br>BMI: 27	Age<br>BMI	GDM<br>GH<br>PE<br>GWG<br>IoL<br>Instrumental<br>CS
Reyes-Munoz et al (2012)	Mexico	Retrospective cohort	Moderate	N: 52<br>Age: 29<br>BMI: 28	N: 52<br>Age: 29<br>BMI: 28	Age<br>BMI<br>Parity	Miscarriage<br>GDM<br>PE<br>GWG
Boutzios et al (2013)	Greece	Prospective	Poor	N: 41<br>Age: 31<br>BMI: 25	N: 110<br>Age: 32<br>BMI: 24	-	GDM
Wang et al (2013)	China	Prospective	High	N: 220<br>Age: 31<br>BMI: 23	N: 652<br>Age: 29<br>BMI: 20	-	Miscarriage<br>GDM<br>GH
Ashrafi et al (2014)	Iran	Retrospective cohort	Poor	N: 234<br>Age: 30<br>BMI: 26	N: 468<br>Age: 29<br>BMI: 26	-	GDM
Elkholi et al (2014)	Egypt	Prospective	Moderate	N: 200<br>Age: 23<br>BMI: 32	N: 200<br>Age: 23<br>BMI: 32	Age<br>BMI<br>SES	Miscarriage<br>GDM<br>GH<br>PE<br>CS
Foroozanfard et al (2014)	Iran	Retrospective cohort	Moderate	N: 130<br>Age: 29<br>BMI: 28	N: 131<br>Age: 29<br>BMI: 28	-	GDM<br>GH<br>PE<br>CS
Huang et al (2014)	China	Retrospective cohort	Moderate	N: 50<br>Age: 30<br>BMI: 23	N: 39<br>Age: 30<br>BMI: 21	-	Miscarriage
Lathi et al (2014)	USA	Prospective	Poor	N: 59<br>Age: 33<br>BMI: 26	N: 287<br>Age: 36<br>BMI: 23	-	Miscarriage
Li et al (2014)	China	Retrospective cohort	Poor	N: 38<br>Age: NR<br>BMI: NR	N: 289<br>Age: NR<br>BMI: NR	-	Miscarriage
Liu et al (2014)	China	Retrospective cohort	Poor	N: 20<br>Age: 31<br>BMI: NR	N: 166<br>Age: 32<br>BMI: NR	-	Miscarriage
Naver et al (2014)	Denmark	Prospective	Poor	N: 459<br>Age: 32<br>BMI: 23	N: 5409<br>Age: 31<br>BMI: 23	-	GDM<br>GH<br>PE<br>IoL<br>CS
Palomba et al (2014)	Italy	Prospective	High	N: 150<br>Age: 28	N: 150<br>Age: 27	Age<br>BMI	Miscarriage<br>GH

				BMI: 27	BMI: 27		
Palomba et al (2014)	Italy	Prospective	High	N: 150<br>Age: 28<br>BMI: NR	N: 150<br>Age: 28<br>BMI: NR	Age<br>BMI	GDM<br>PE<br>Instrumental<br>CS
Zhang et al (2014)	China	Prospective	Poor	N: 27<br>Age: 30<br>BMI: 24	N: 27<br>Age: 30<br>BMI: 23	-	Miscarriage
Kollmann et al (2015)	Austria	Retrospective cohort	Poor	N: 177<br>Age: NR<br>BMI: NR	N: 708<br>Age: NR<br>BMI: NR	-	GDM<br>GH<br>PE<br>Instrumental<br>CS
Koster et al (2015)	Netherlands	Prospective	Poor	N: 73<br>Age: 31<br>BMI: NR	N: 209<br>Age: 32<br>BMI: NR	-	GDM<br>IoL<br>CS
Mumm et al (2015)	Denmark	Prospective	Poor	N: 157<br>Age: NR<br>BMI: NR	N: 1037<br>Age: NR<br>BMI: NR	-	GDM<br>GH<br>PE<br>IoL<br>Instrumental<br>CS
Sawada et al (2015)	Japan	Retrospective cohort	High	N: 49<br>Age: 32<br>BMI: 24	N: 49<br>Age: 32<br>BMI: 24	Age<br>BMI<br>Parity	GDM<br>GH<br>CS
Wan et al (2015)	China	Retrospective cohort	Moderate	N: 24<br>Age: 31<br>BMI: 23	N: 224<br>Age: 33<br>BMI: 21	Age	GDM<br>GH<br>PE<br>EC
Aktun et al (2016)	Turkey	Prospective	Poor	N: 150<br>Age: 29<br>BMI: 23	N: 160<br>Age: 31<br>BMI: 21	-	GH<br>PE<br>GWG<br>CS
Sterling et al (2016)	Canada	Retrospective cohort	Poor	N: 71<br>Age: NR<br>BMI: 25	N: 323<br>Age: NR<br>BMI: 24	-	GDM<br>CS
Wang et al (2016)	China	Retrospective cohort	Poor	N: 1361<br>Age: 30<br>BMI: 24	N: 15921<br>Age: 33<br>BMI: 23	-	Miscarriage
Wang et al (2016)	China	Prospective	Poor	N: 119<br>Age: 32<br>BMI: NR	N: 664<br>Age: 33<br>BMI: NR	-	Miscarriage
Xiao et al (2016)	China	Retrospective cohort	Poor	N: 352<br>Age: 30<br>BMI: NR	N: 2037<br>Age: 29<br>BMI: NR	-	GDM<br>CS
Chen et al (2017)	China	Prospective	Poor	N: 22<br>Age: NR<br>BMI: NR	N: 73<br>Age: NR<br>BMI: NR	-	Miscarriage
deWilde et al (2017)	Netherlands	Prospective	Poor	N: 188<br>Age: NR<br>BMI: NR	N: 2889<br>Age: NR<br>BMI: NR	-	GDM<br>GH<br>PE<br>IoL<br>CS
Jonsdottir et al (2017)	Denmark	Retrospective cohort	Moderate	N: 72<br>Age: 33<br>BMI: 22	N: 288<br>Age: 32<br>BMI: 23	-	GDM<br>PE<br>IoL<br>CS
Luo et al (2017)	China	Retrospective cohort	High	N: 34<br>Age: 30<br>BMI: 21	N: 111<br>Age: 31<br>BMI: 22	Age<br>BMI	Miscarriage
Lai et al (2018)	China	Prospective	Poor	N: 22<br>Age: NR<br>BMI: NR	N: 25<br>Age: NR<br>BMI: NR	-	Miscarriage
Huang et al (2018)	China	Retrospective cohort	Poor	N: 89<br>Age: NR<br>BMI: NR	N: 249<br>Age: NR<br>BMI: NR	-	Miscarriage

Kent et al (2018)	USA	Retrospective cohort	Moderate	N: 164<br>Age: 28<br>BMI: 33	N: 154<br>Age: 32<br>BMI: 28	-	GDM<br>PE
Li et al (2018)	China	Retrospective cohort	Poor	N: 670<br>Age: 31<br>BMI: 24	N: 6000<br>Age: 32<br>BMI: 22	-	Miscarriage<br>GDM<br>GH
Butts et al (2019)	USA	Retrospective cohort	Poor	N: 205<br>Age: 29<br>BMI: 36	N: 228<br>Age: 32<br>BMI: 27	-	Miscarriage
Kollmann et al (2019)	Austria	Retrospective cohort	Moderate	N: 79<br>Age: 31<br>BMI: 30	N: 354<br>Age: 30<br>BMI: 29	-	GDM<br>GH<br>PE
Schneider et al (2019)	USA	Retrospective cohort	Poor	N: 809<br>Age: 31<br>BMI: NR	N: 956<br>Age: 31<br>BMI: NR	-	GH
Zheng et al (2019)	China	Retrospective cohort	High	N: 242<br>Age: 30<br>BMI: 24	N: 324<br>Age: 31<br>BMI: 25	-	GDM<br>GH<br>PE
Benito et al (2020)	Spain	Prospective	Moderate	N: 20<br>Age: 34<br>BMI: 29	N: 30<br>Age: 34<br>BMI: 29	-	Miscarriage<br>PE<br>IoL<br>Instrumental<br>CS
Chen et al (2020)	China	Prospective	Poor	N: 35<br>Age: NR<br>BMI: NR	N: 29<br>Age: NR<br>BMI: NR	-	Miscarriage<br>CS
Elshewyt et al (2020)	China	Prospective	Poor	N: 33<br>Age: NR<br>BMI: NR	N: 35<br>Age: NR<br>BMI: NR	-	Miscarriage
Foroozanfard et al (2020)	Iran	Prospective	Poor	N: 41<br>Age: 25<br>BMI: 26	N: 47<br>Age: 26<br>BMI: 24	-	Miscarriage<br>GDM<br>PE<br>CS
Liu et al (2020)	China	Retrospective cohort	Poor	N: 472<br>Age: NR<br>BMI: NR	N: 4190<br>Age: NR<br>BMI: NR	-	Miscarriage<br>GDM<br>GH<br>CS
Tobiasz et al (2020)	USA	Prospective	Poor	N: 17<br>Age: NR<br>BMI: NR	N: 11<br>Age: NR<br>BMI: NR	-	CS
Abdulkhalikova et al (2021)	Slovenia	Retrospective cohort	Poor	N: 73<br>Age: 33<br>BMI: NR	N: 196<br>Age: 34<br>BMI: NR	-	GDM<br>GH<br>PE<br>GWG<br>CS
Cai et al (2021)	China	Retrospective cohort	Moderate	N: 2357<br>Age: 29<br>BMI: 24	N: 19463<br>Age: 31<br>BMI: 22	-	Miscarriage<br>GDM
Feichtinger et al (2021)	Austria	Prospective	Poor	N: 31<br>Age: NR<br>BMI: NR	N: 36<br>Age: NR<br>BMI: NR	-	GDM
Gongadashetti et al (2021)	India	Prospective	Poor	N: 12<br>Age: NR<br>BMI: NR	N: 14<br>Age: NR<br>BMI: NR	-	Miscarriage
Hu et al (2021)	China	Retrospective cohort	Poor	N: 557<br>Age: 30<br>BMI: 23	N: 3526<br>Age: 31<br>BMI: 22	-	Miscarriage<br>GDM<br>CS
Jiang et al (2021)	China	Retrospective cohort	Poor	N: 30<br>Age: 31<br>BMI: 25	N: 31<br>Age: 32<br>BMI: 23	-	GDM<br>GH<br>Instrumental<br>CS
Kollmann et al (2021)	Austria	Retrospective cohort	Moderate	N: 79<br>Age: 31<br>BMI: 30	N: 354<br>Age: 30<br>BMI: 29	-	GDM<br>GH<br>PE
Liu et al (2021)	China	Retrospective cohort	Poor	N: 92<br>Age: 30	N: 112<br>Age: 30	-	CS

				BMI: 24	BMI: 23		
Liu et al (2021)	China	Prospective	Poor	N: 86<br>Age: 28<br>BMI: 24	N: 60<br>Age: 28<br>BMI: 22	-	Miscarriage
Mai et al (2021)	China	Retrospective cohort	Poor	N: 192<br>Age: NR<br>BMI: NR	N: 298<br>Age: NR<br>BMI: NR	-	Miscarriage
March et al (2021)	Australia	Retrospective cohort	Moderate	N: 52<br>Age: NR<br>BMI: NR	N: 514<br>Age: NR<br>BMI: NR	-	Depression
Pouya et al (2021)	Turkey	Retrospective cohort	Poor	N: 88<br>Age: 29<br>BMI: 25	N: 90<br>Age: 32<br>BMI: 24	-	Miscarriage
Wang et al (2021)	China	Retrospective cohort	Moderate	N: 29<br>Age: NR<br>BMI: 22	N: 116<br>Age: NR<br>BMI: 21	-	GWG<br>CS
Wu et al (2021)	China	Retrospective cohort	High	N: 23<br>Age: 30<br>BMI: 23	N: 11<br>Age: 30<br>BMI: 22	Age<br>BMI<br>Parity	Miscarriage
Zhu et al (2021)	China	Retrospective cohort	Poor	N: 111<br>Age: NR<br>BMI: NR	N: 237<br>Age: NR<br>BMI: NR	-	Miscarriage<br>GDM<br>GH
Tu et al (2022)	China	Retrospective cohort	Poor	N: 27<br>Age: 29<br>BMI: 22	N: 22<br>Age: 30<br>BMI: 21	-	Miscarriage
Liu et al (2022)	China	Retrospective cohort	Poor	N: 1357<br>Age: 31<br>BMI: NR	N: 6940<br>Age: 32<br>BMI: NR	-	GDM<br>GH<br>PE
Ni t al (2022)	China	Retrospective cohort	Poor	N: 1376<br>Age: 31<br>BMI: 24	N: 1376<br>Age: 31<br>BMI: 22	-	Miscarriage
Song et al (2022)	China	Retrospective cohort	Poor	N: 70<br>Age: 29<br>BMI: 22	N: 105<br>Age30<br>BMI: 22	Ethnicity	Miscarriage
Wang et al (2022)	China	Retrospective cohort	Poor	N: 346<br>Age: 29<br>BMI: 25	N: 453<br>Age: 29<br>BMI: 23	-	GDM<br>CS
Yang et al (2022)	China	Retrospective cohort	Poor	N: 208<br>Age: 28<br>BMI: 22	N: 1506<br>Age: 30<br>BMI: 21	-	Miscarriage

BMI: body mass index; CS: caesarean section; EC: eclampsia; GDM: gestational diabetes; GH: gestational hypertension; GWG: gestational weight gain; IoL: induction of labour; NR: not reported; PCOS: polycystic ovary syndrome; PE: pre-eclampsia; SES: socioeconomic status

*Age is reported in years

†BMI is reported in kg/m^2

Supplementary Figure 1_a. Association of polycystic ovary syndrome with gestational weight gain.

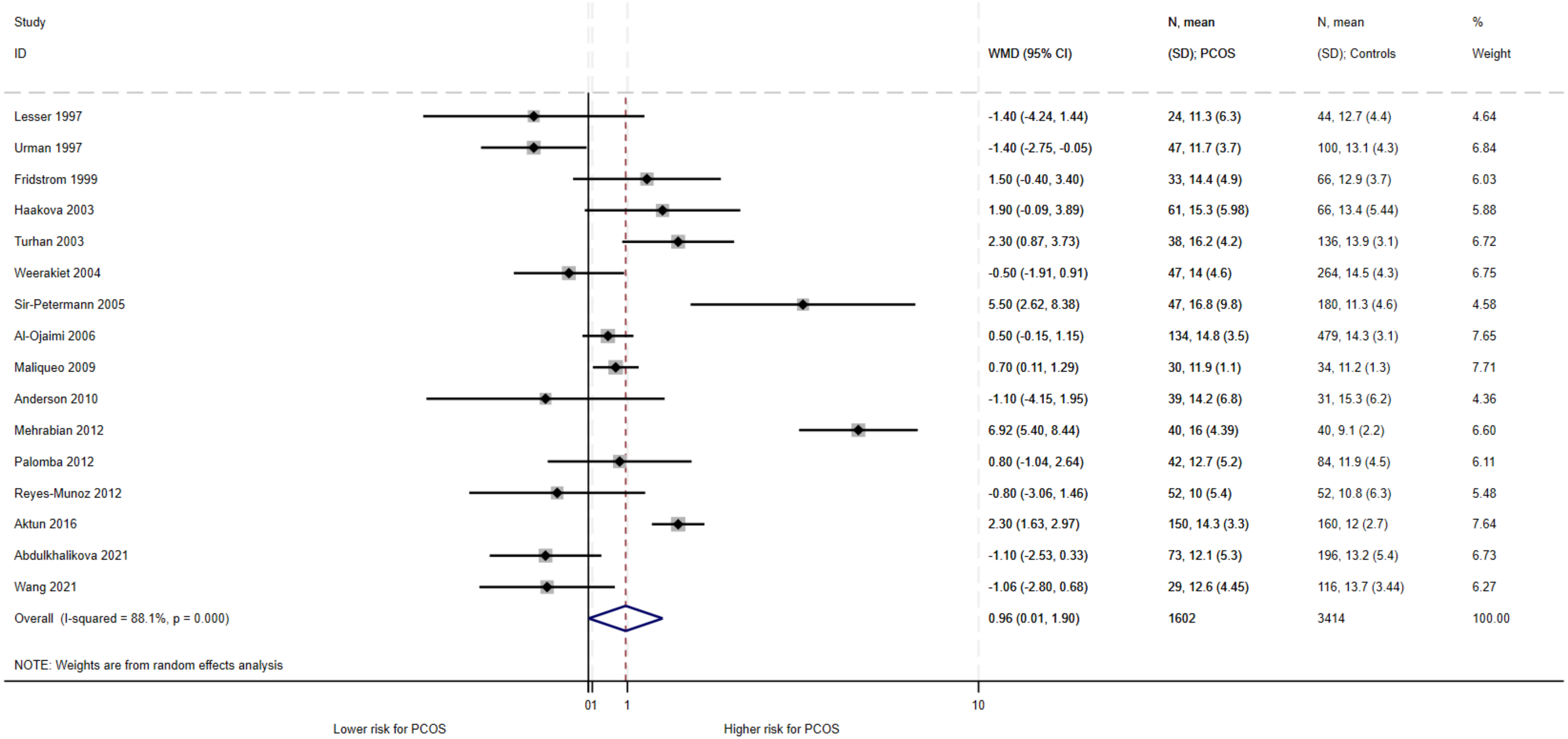

Supplementary Figure 1_b. Cumulative plot of association of polycystic ovary syndrome with gestational weight gain

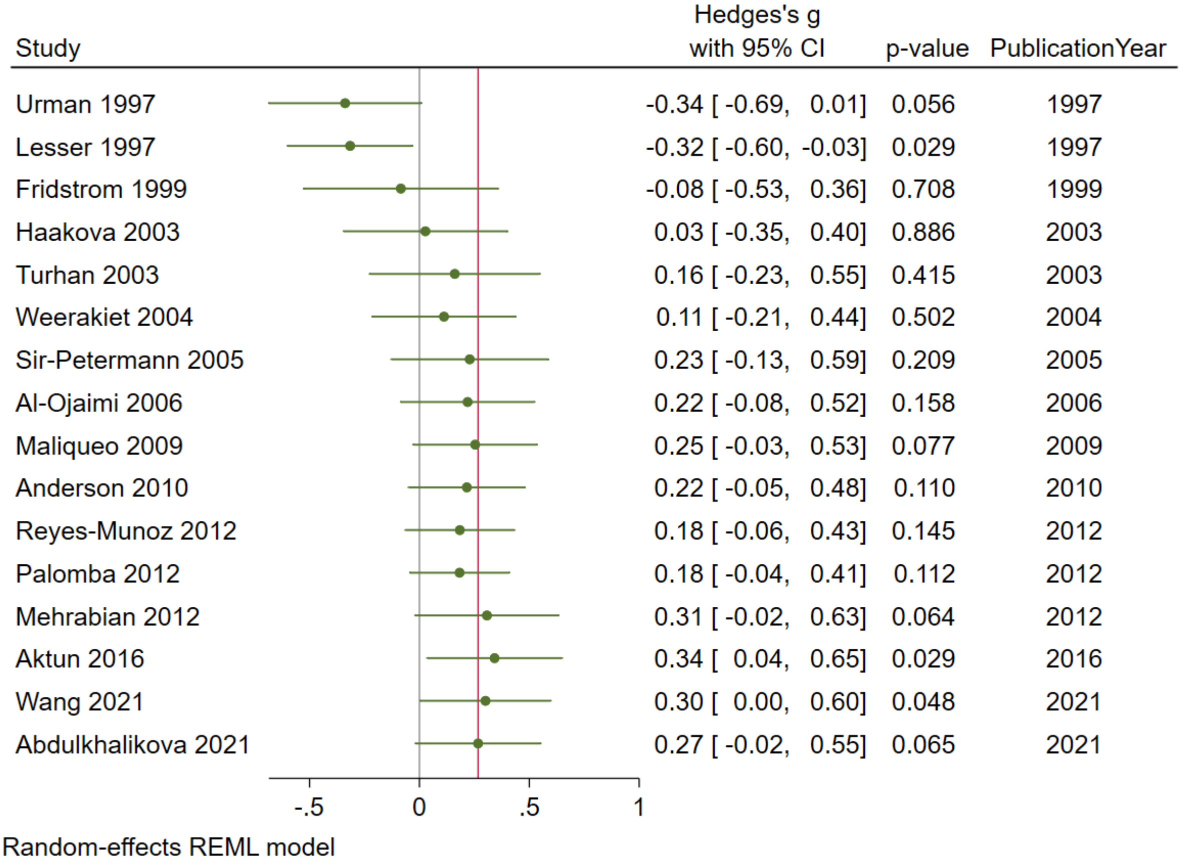

Supplementary Figure 1_c. Funnel plot assessing publication bias in studies on the association of polycystic ovary syndrome with gestational weight gain

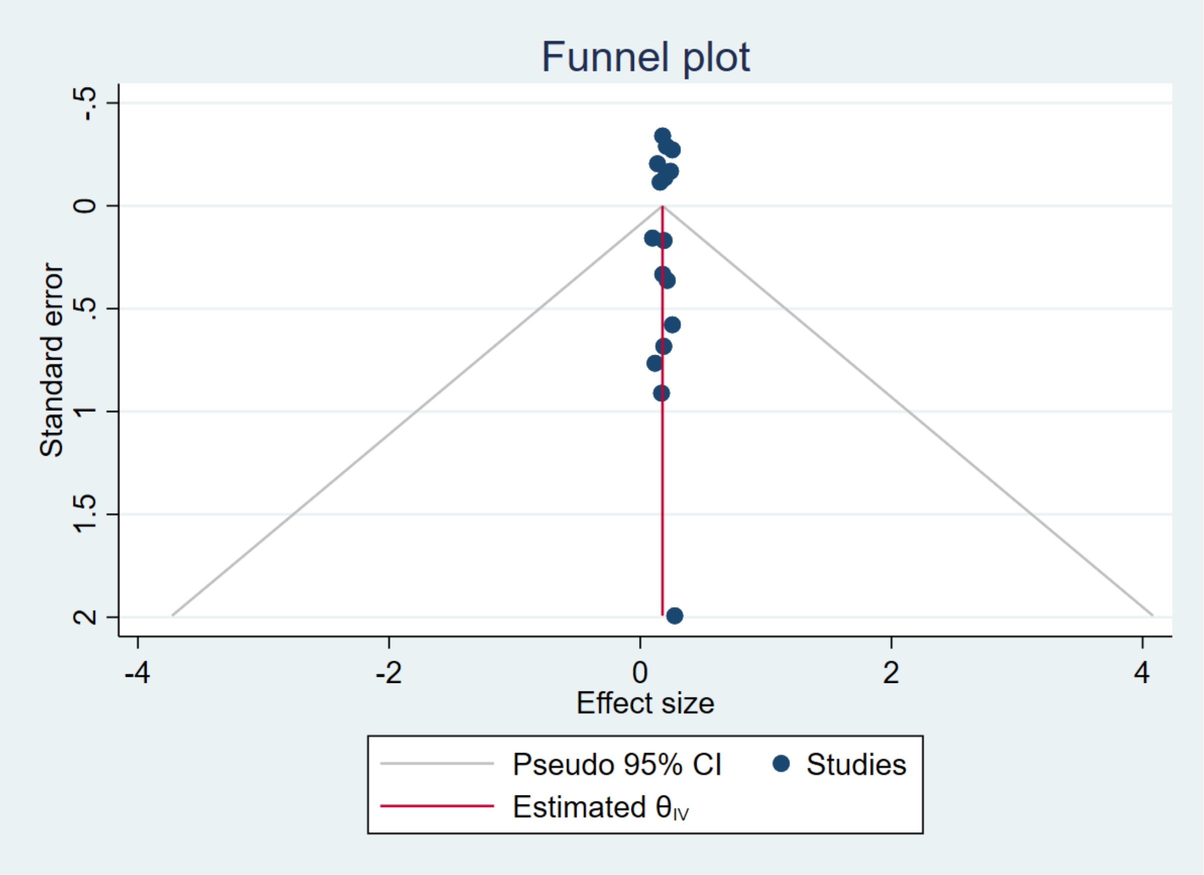

Source data are provided as a Source Data file.

Egger's test for small study effects on the outcome of gestational weight gain

Number of studies = 16 Root MSE = 3.022

Std_Eff	Coefficient	Std. err.	t	P>|t|	[95% conf. interval]	
slope	0.1560978	0.4422178	0.35	0.729	-0.7923651	1.104561
bias	0.5641875	2.605411	0.22	0.832	-5.023864	6.152239

Test of H0: no small-study effects P = 0.832

Supplementary Figure 2_a. Forest Plot of association of polycystic ovary syndrome with miscarriage

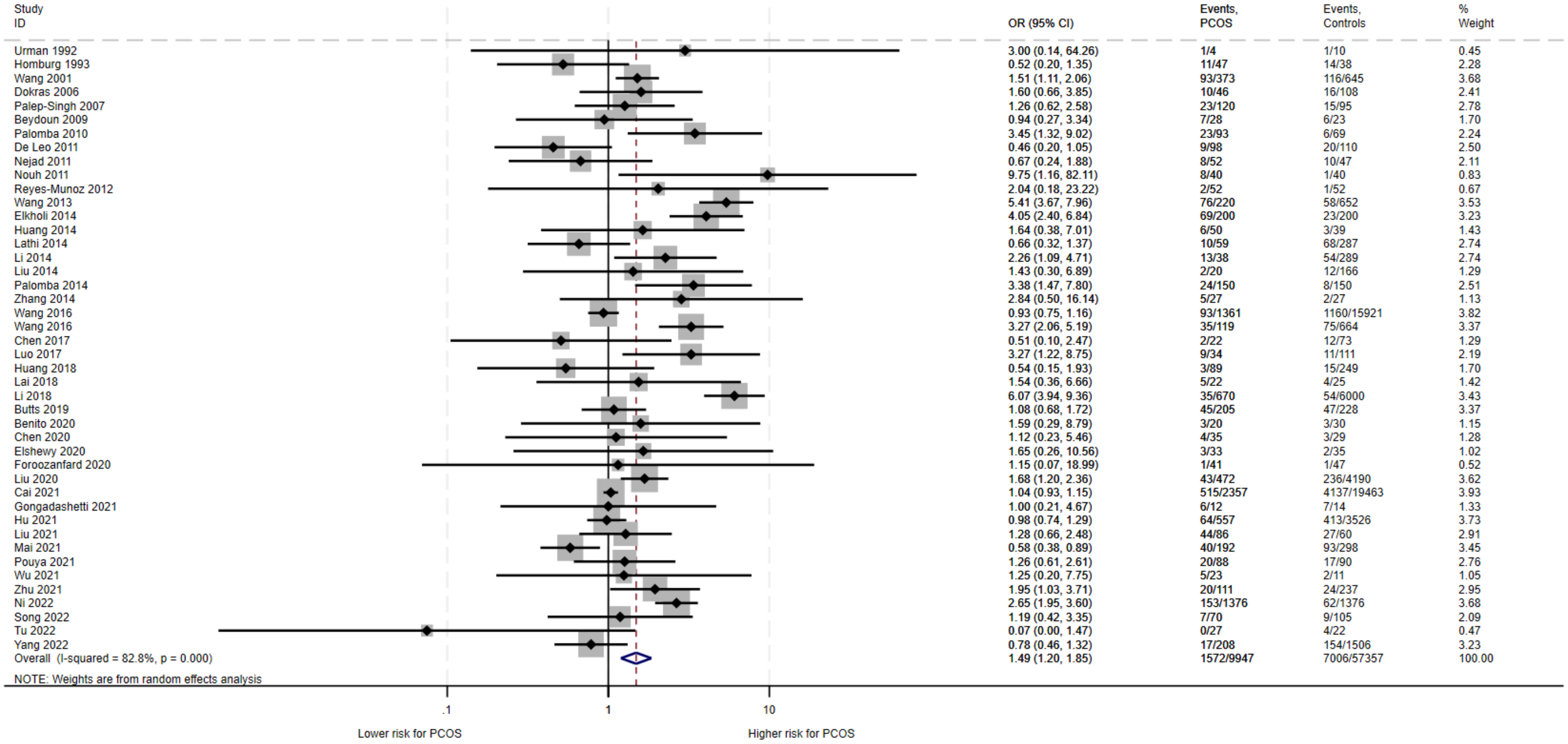

Supplementary Figure 2_b. Cumulative plot of association of polycystic ovary syndrome with miscarriage

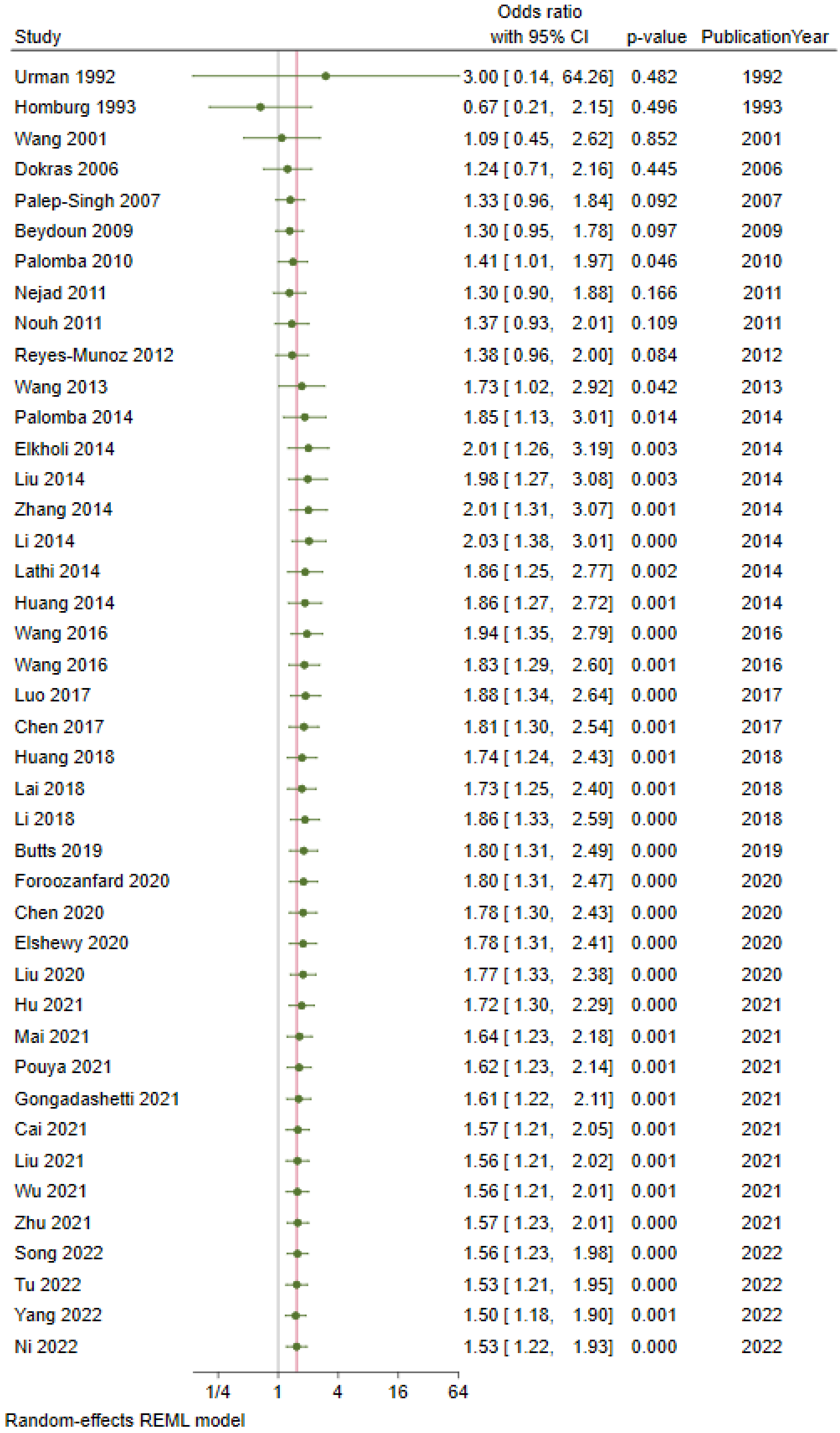

Supplementary Figure 2_c. Funnel plot assessing publication bias in studies on the association of polycystic ovary syndrome with miscarriage

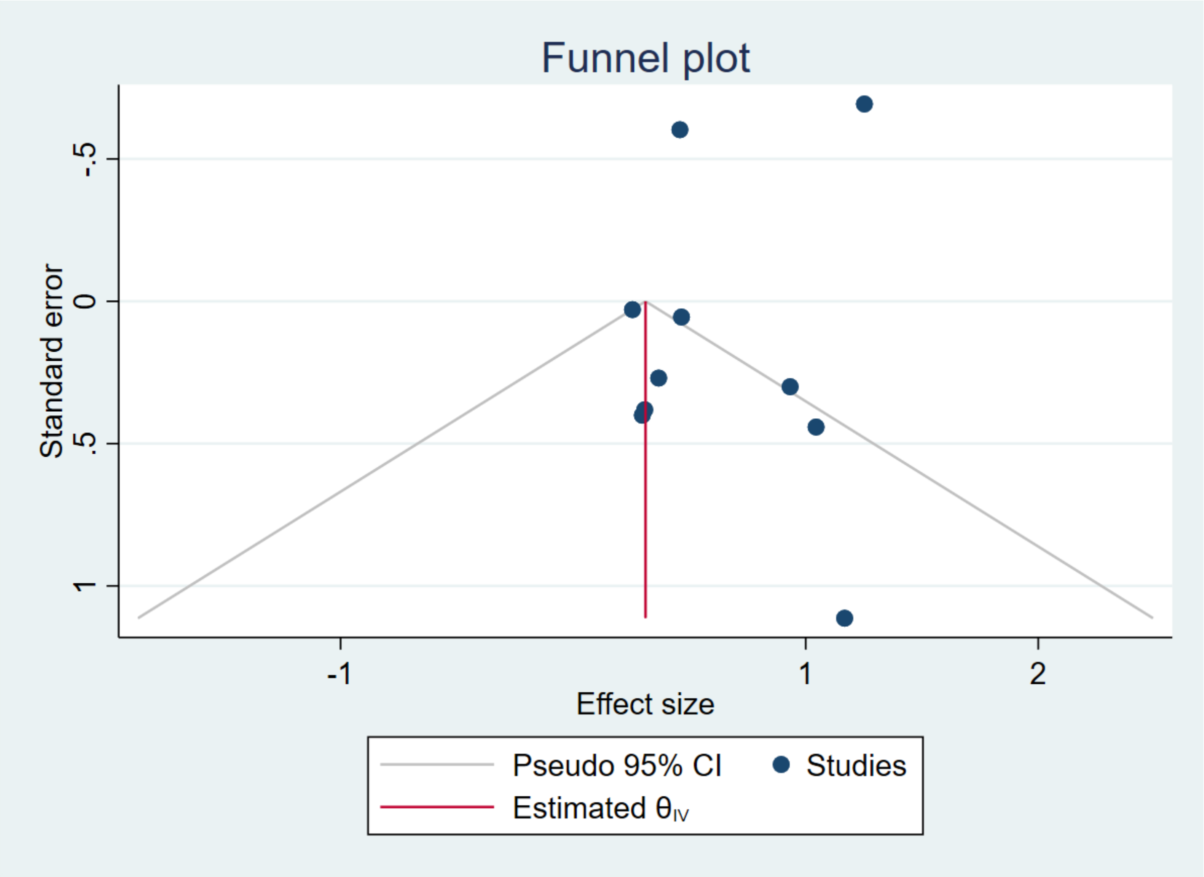

Source data are provided as a Source Data file.

Egger's test for small study effects on the outcome of miscarriage

Number of studies = 44 Root MSE = 2.392

Std_Eff	Coefficient	Std. err.	t	P>|t|	[95% conf. interval]	
slope	0.1499098	0.1170628	1.28	0.207	-0.0863325	0.3861522
bias	0.6758118	0.5111127	1.32	0.193	-0.3556555	1.707279

Test of H0: no small-study effects P = 0.193

Supplementary Figure 3_a. Forest Plot of association of polycystic ovary syndrome with gestational diabetes

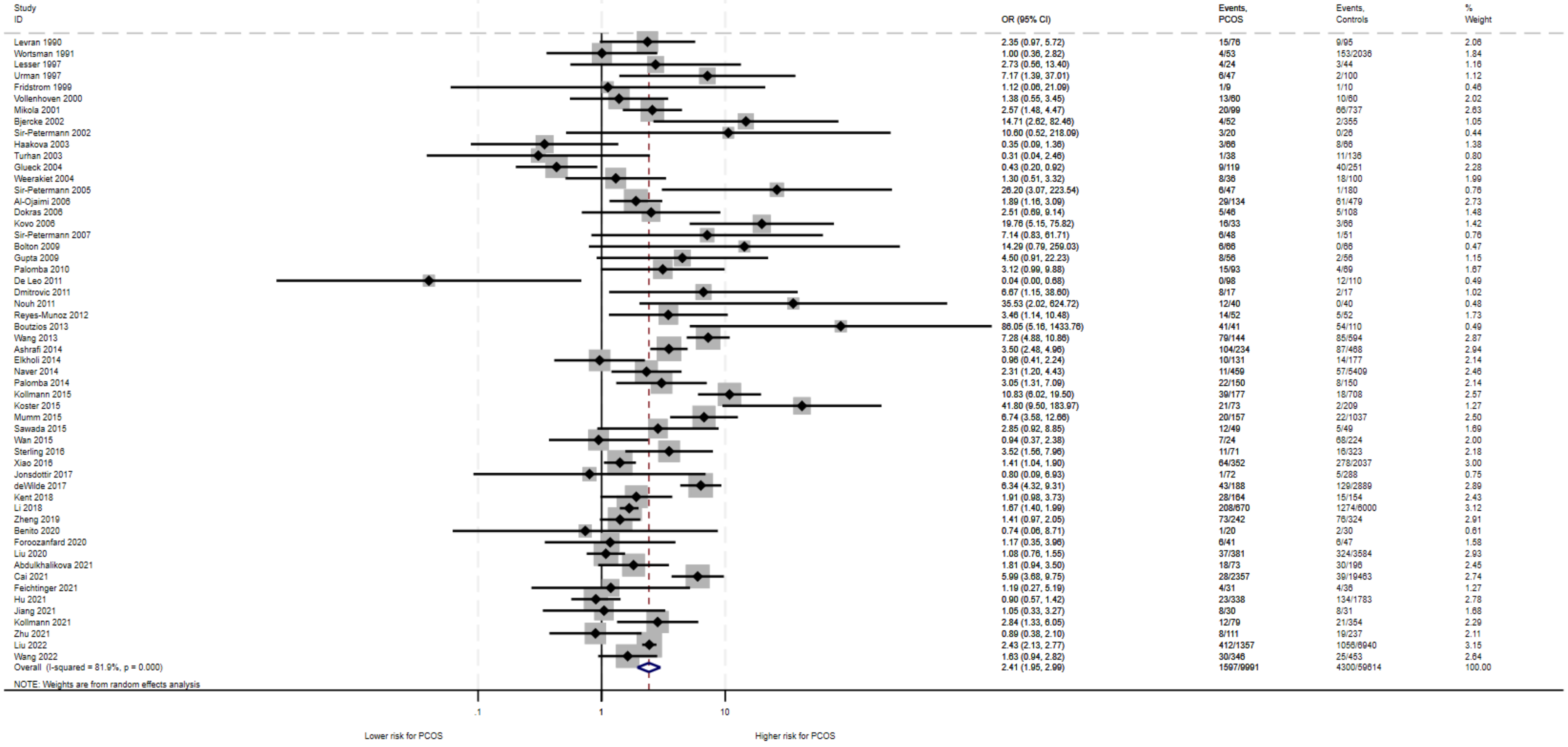

Supplementary Figure 3_b. Cumulative plot of association of polycystic ovary syndrome with gestational diabetes

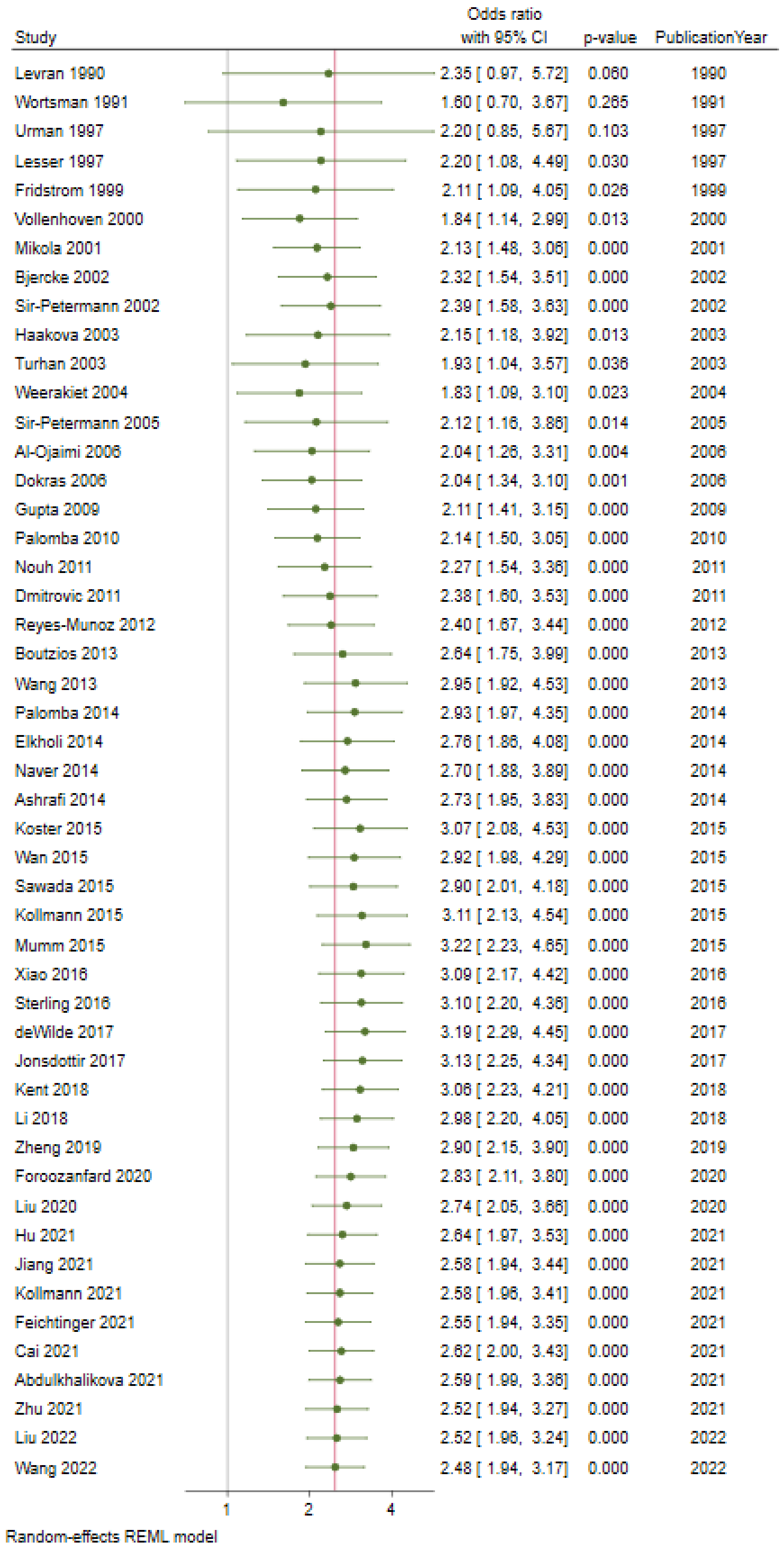

Supplementary Figure 3_c. Funnel plot assessing publication bias in studies on the association of polycystic ovary syndrome with gestational diabetes

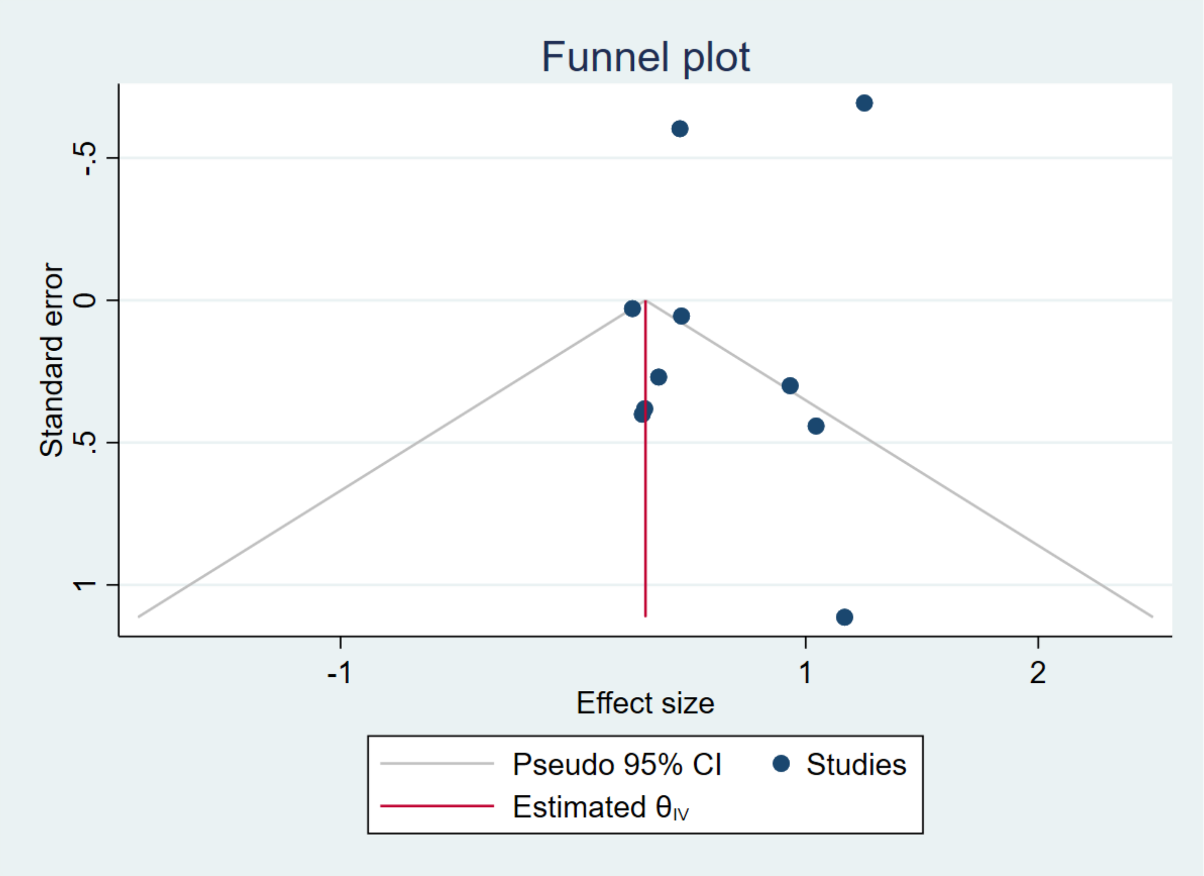

Source data are provided as a Source Data file.

Egger's test for small study effects on the outcome of gestational diabetes

Number of studies = 55 Root MSE = 2.359

Std_Eff	Coefficient	Std. err.	t	P>|t|	[95% conf. interval]	
slope	0.7423486	0.1276175	5.82	0.000	0.4863803	0.9983169
bias	0.3398212	0.4690604	0.72	0.472	-0.6009948	1.280637

Test of H0: no small-study effects P = 0.472

Supplementary Figure 4_a. Forest Plot of association of polycystic ovary syndrome with gestational hypertension

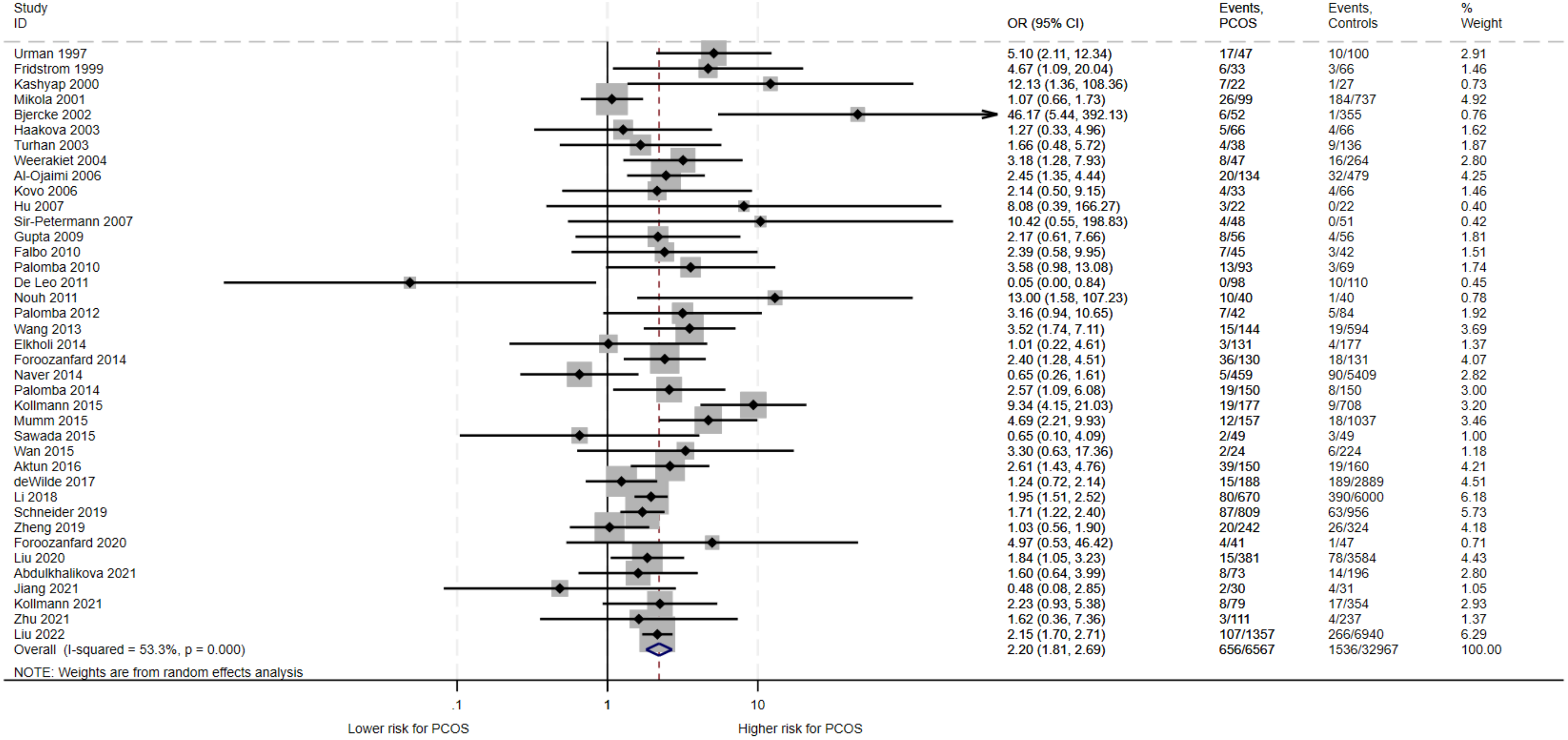

Study ID	OR (95% CI)	Events, PCOS	Events, Controls	% Weight
Urman 1997	5.10 (2.11, 12.34)	17/47	10/100	2.91
Fridstrom 1999	4.67 (1.09, 20.04)	6/33	3/66	1.46
Kashyap 2000	12.13 (1.36, 108.36)	7/22	1/27	0.73
Mikola 2001	1.07 (0.66, 1.73)	26/99	184/737	4.92
Bjercke 2002	46.17 (5.44, 392.13)	6/52	1/355	0.76
Haakova 2003	1.27 (0.33, 4.96)	5/66	4/66	1.62
Turhan 2003	1.66 (0.48, 5.72)	4/38	9/136	1.87
Weerakiet 2004	3.18 (1.28, 7.93)	8/47	16/264	2.80
Al-Ojaimi 2006	2.45 (1.35, 4.44)	20/134	32/479	4.25
Kovo 2006	2.14 (0.50, 9.15)	4/33	4/66	1.46
Hu 2007	8.08 (0.39, 166.27)	3/22	0/22	0.40
Sir-Petermann 2007	10.42 (0.55, 198.83)	4/48	0/51	0.42
Gupta 2009	2.17 (0.61, 7.66)	8/56	4/56	1.81
Falbo 2010	2.39 (0.58, 9.95)	7/45	3/42	1.51
Palomba 2010	3.58 (0.98, 13.08)	13/93	3/69	1.74
De Leo 2011	0.05 (0.00, 0.84)	0/98	10/110	0.45
Nouh 2011	13.00 (1.58, 107.23)	10/40	1/40	0.78
Palomba 2012	3.16 (0.94, 10.65)	7/42	5/84	1.92
Wang 2013	3.52 (1.74, 7.11)	15/144	19/594	3.69
Elkholi 2014	1.01 (0.22, 4.61)	3/131	4/177	1.37
Foroozanfard 2014	2.40 (1.28, 4.51)	36/130	18/131	4.07
Naver 2014	0.65 (0.26, 1.61)	5/459	90/5409	2.82
Palomba 2014	2.57 (1.09, 6.08)	19/150	8/150	3.00
Kollmann 2015	9.34 (4.15, 21.03)	19/177	9/708	3.20
Mumm 2015	4.69 (2.21, 9.93)	12/157	18/1037	3.46
Sawada 2015	0.65 (0.10, 4.09)	2/49	3/49	1.00
Wan 2015	3.30 (0.63, 17.36)	2/24	6/224	1.18
Aktun 2016	2.61 (1.43, 4.76)	39/150	19/160	4.21
deWilde 2017	1.24 (0.72, 2.14)	15/188	189/2889	4.51
Li 2018	1.95 (1.51, 2.52)	80/670	390/6000	6.18
Schneider 2019	1.71 (1.22, 2.40)	87/809	63/956	5.73
Zheng 2019	1.03 (0.56, 1.90)	20/242	26/324	4.18
Foroozanfard 2020	4.97 (0.53, 46.42)	4/41	1/47	0.71
Liu 2020	1.84 (1.05, 3.23)	15/381	78/3584	4.43
Abdulkhalikova 2021	1.60 (0.64, 3.99)	8/73	14/196	2.80
Jiang 2021	0.48 (0.08, 2.85)	2/30	4/31	1.05
Kollmann 2021	2.23 (0.93, 5.38)	8/79	17/354	2.93
Zhu 2021	1.62 (0.36, 7.36)	3/111	4/237	1.37
Liu 2022	2.15 (1.70, 2.71)	107/1357	266/6940	6.29
Overall (I-squared = 53.3%, p = 0.000)	2.20 (1.81, 2.69)	656/6567	1536/32967	100.00

NOTE: Weights are from random effects analysis

Supplementary Figure 4_b. Cumulative plot of association of polycystic ovary syndrome with gestational hypertension

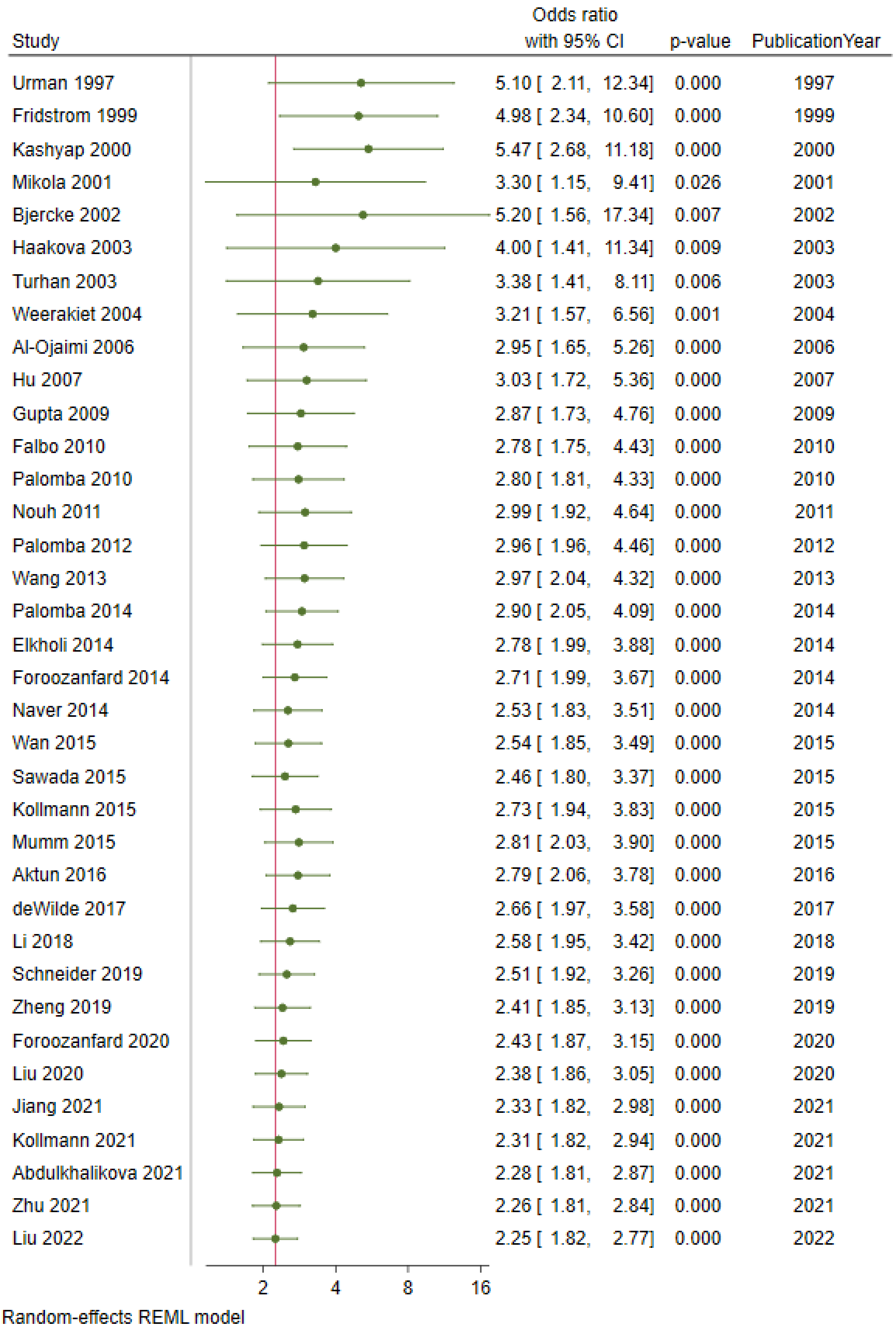

Supplementary Figure 4_c. Funnel plot assessing publication bias in studies on the association of polycystic ovary syndrome with gestational hypertension

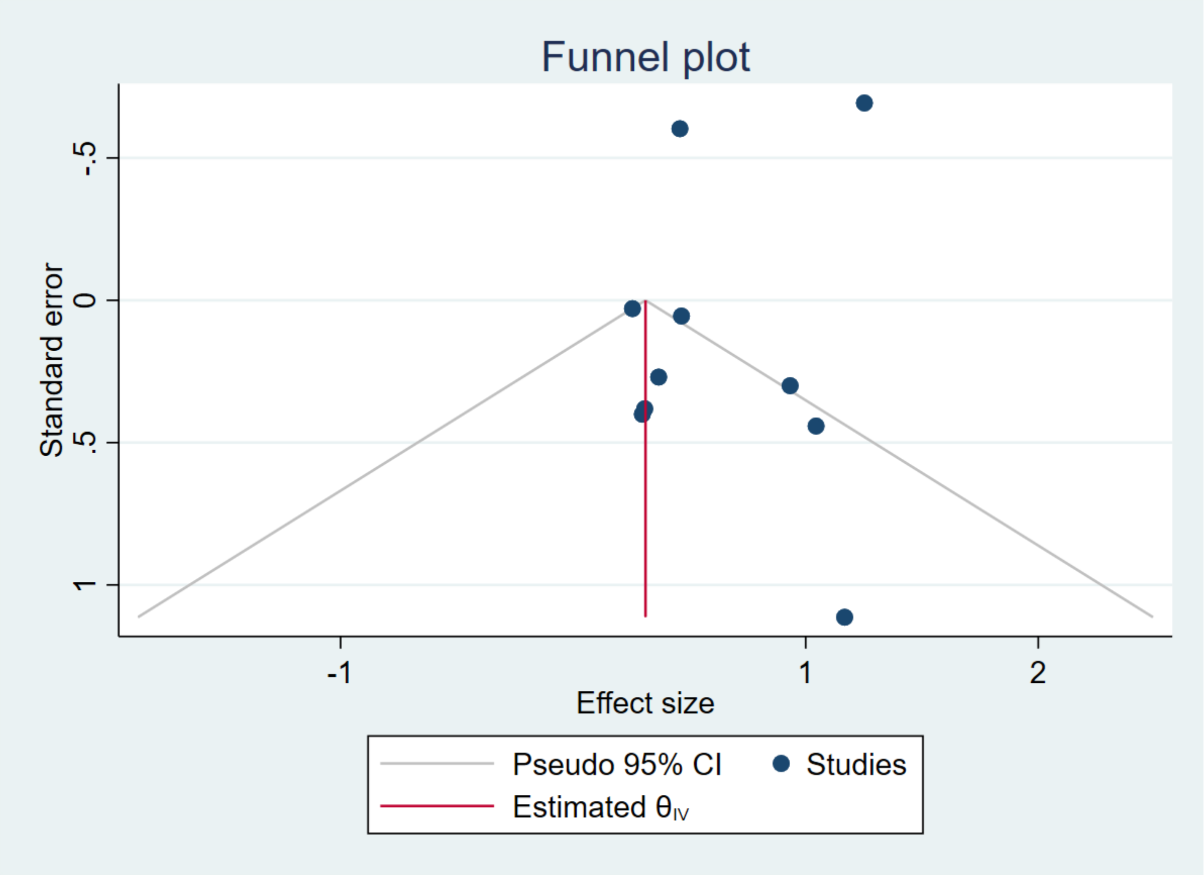

Source data are provided as a Source Data file.

Egger's test for small study effects on the outcome of gestational hypertension

Number of studies = 39 Root MSE = 1.451

Std_Eff	Coefficient	Std. err.	t	P>|t|	[95% conf. interval]	
slope	0.5824468	0.1344852	4.33	0.000	0.309954	0.8549396
bias	0.4825391	0.3885063	1.24	0.222	-.3046494	1.269728

Test of H0: no small-study effects P = 0.222

Supplementary Figure 5_a. Forest Plot of association of polycystic ovary syndrome with pre-eclampsia

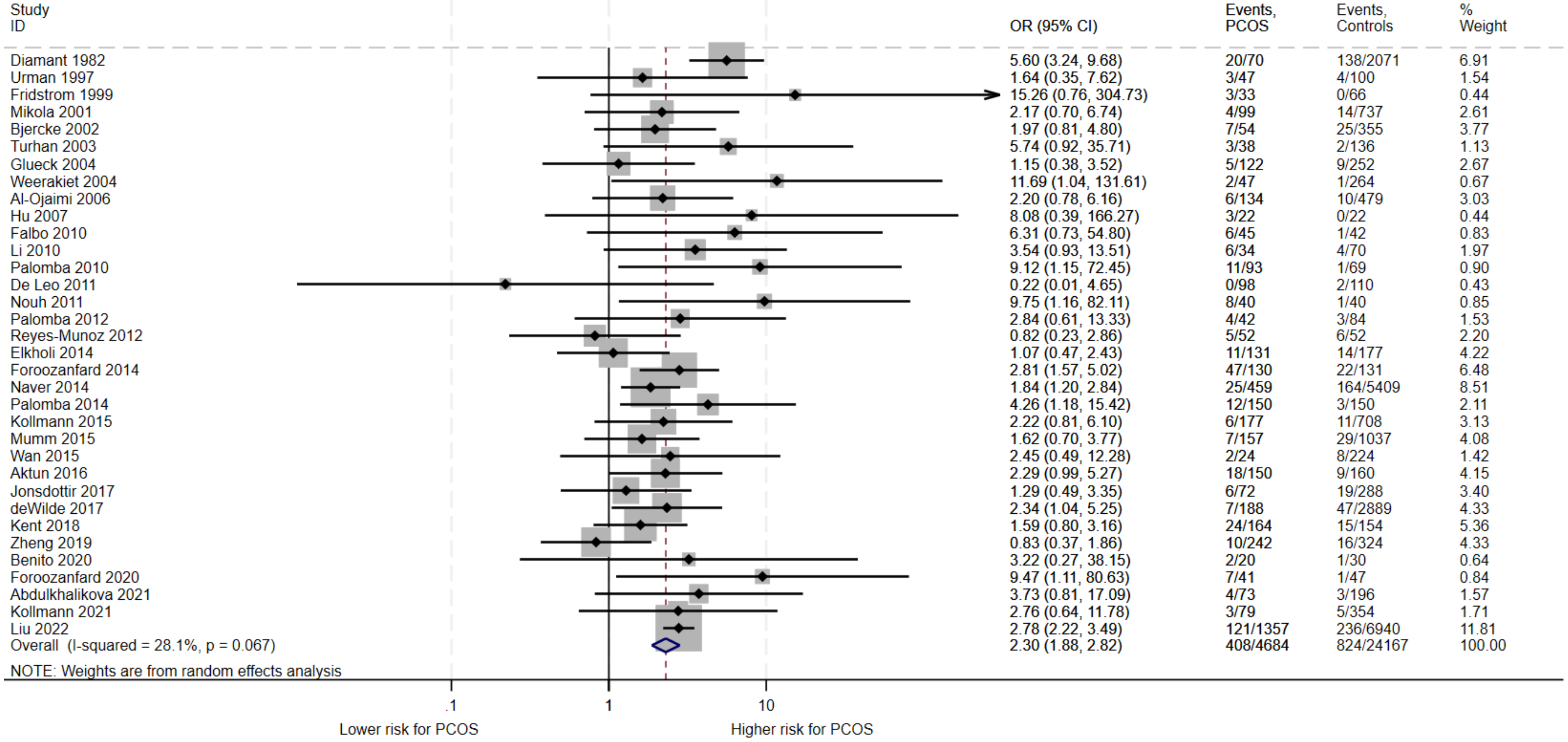

Study ID	OR (95% CI)	Events, PCOS	Events, Controls	% Weight
Diamant 1982	5.60 (3.24, 9.68)	20/70	138/2071	6.91
Urman 1997	1.64 (0.35, 7.62)	3/47	4/100	1.54
Fridstrom 1999	15.26 (0.76, 304.73)	3/33	0/66	0.44
Mikola 2001	2.17 (0.70, 6.74)	4/99	14/737	2.61
Bjercke 2002	1.97 (0.81, 4.80)	7/54	25/355	3.77
Turhan 2003	5.74 (0.92, 35.71)	3/38	2/136	1.13
Glueck 2004	1.15 (0.38, 3.52)	5/122	9/252	2.67
Weerakiet 2004	11.69 (1.04, 131.61)	2/47	1/264	0.67
Al-Ojaimi 2006	2.20 (0.78, 6.16)	6/134	10/479	3.03
Hu 2007	8.08 (0.39, 166.27)	3/22	0/22	0.44
Falbo 2010	6.31 (0.73, 54.80)	6/45	1/42	0.83
Li 2010	3.54 (0.93, 13.51)	6/34	4/70	1.97
Palomba 2010	9.12 (1.15, 72.45)	11/93	1/69	0.90
De Leo 2011	0.22 (0.01, 4.65)	0/98	2/110	0.43
Nouh 2011	9.75 (1.16, 82.11)	8/40	1/40	0.85
Palomba 2012	2.84 (0.61, 13.33)	4/42	3/84	1.53
Reyes-Munoz 2012	0.82 (0.23, 2.86)	5/52	6/52	2.20
Elkholi 2014	1.07 (0.47, 2.43)	11/131	14/177	4.22
Foroozanfard 2014	2.81 (1.57, 5.02)	47/130	22/131	6.48
Naver 2014	1.84 (1.20, 2.84)	25/459	164/5409	8.51
Palomba 2014	4.26 (1.18, 15.42)	12/150	3/150	2.11
Kollmann 2015	2.22 (0.81, 6.10)	6/177	11/708	3.13
Mumm 2015	1.62 (0.70, 3.77)	7/157	29/1037	4.08
Wan 2015	2.45 (0.49, 12.28)	2/24	8/224	1.42
Aktun 2016	2.29 (0.99, 5.27)	18/150	9/160	4.15
Jonsdottir 2017	1.29 (0.49, 3.35)	6/72	19/288	3.40
deWilde 2017	2.34 (1.04, 5.25)	7/188	47/2889	4.33
Kent 2018	1.59 (0.80, 3.16)	24/164	15/154	5.36
Zheng 2019	0.83 (0.37, 1.86)	10/242	16/324	4.33
Benito 2020	3.22 (0.27, 38.15)	2/20	1/30	0.64
Foroozanfard 2020	9.47 (1.11, 80.63)	7/41	1/47	0.84
Abdulkhalikova 2021	3.73 (0.81, 17.09)	4/73	3/196	1.57
Kollmann 2021	2.76 (0.64, 11.78)	3/79	5/354	1.71
Liu 2022	2.78 (2.22, 3.49)	121/1357	236/6940	11.81
Overall (I-squared = 28.1%, p = 0.067)	2.30 (1.88, 2.82)	408/4684	824/24167	100.00

NOTE: Weights are from random effects analysis

Supplementary Figure 5_b. Cumulative plot of association of polycystic ovary syndrome with pre-eclampsia

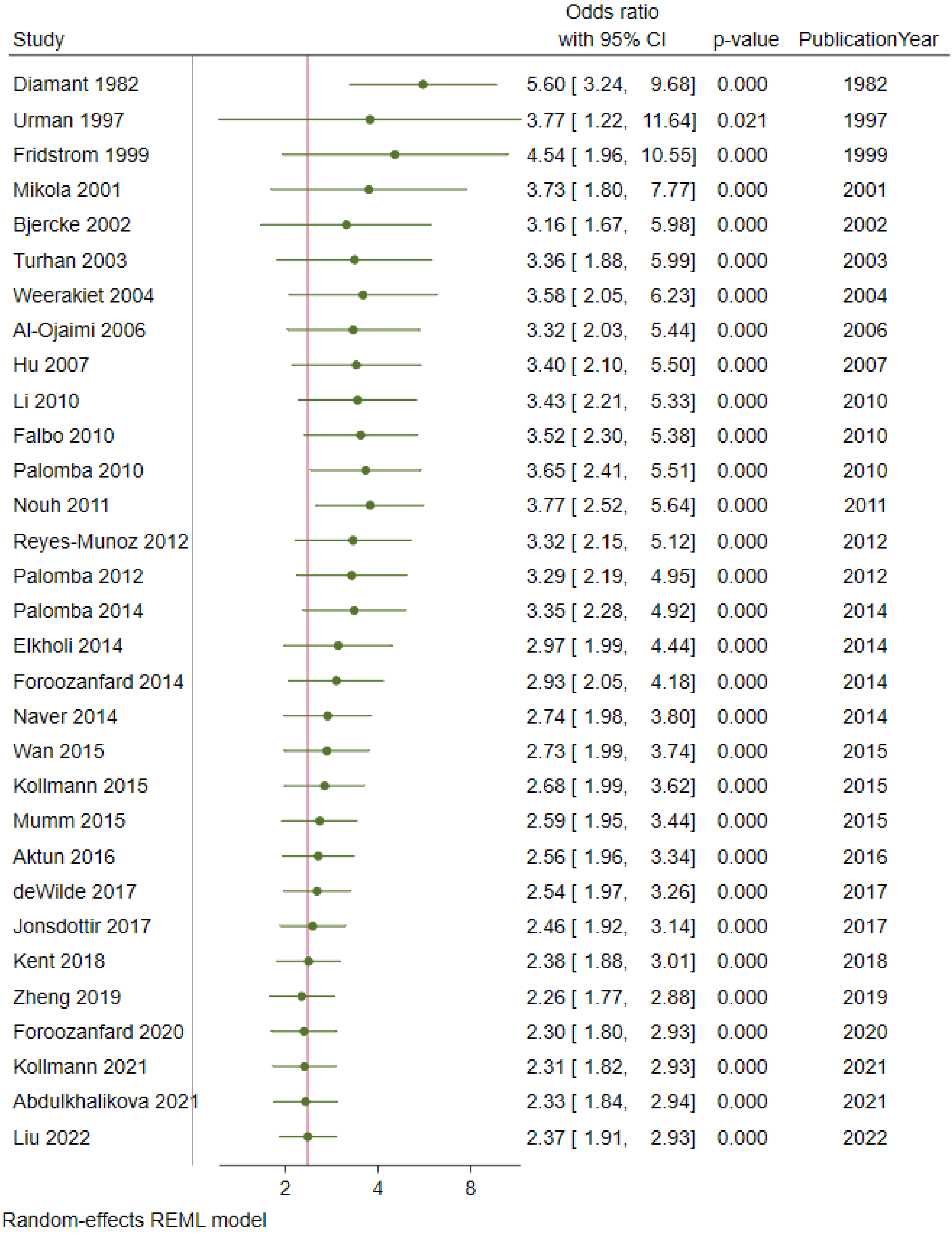

Supplementary Figure 5_c. Funnel plot assessing publication bias in studies on the association of polycystic ovary syndrome with pre-eclampsia

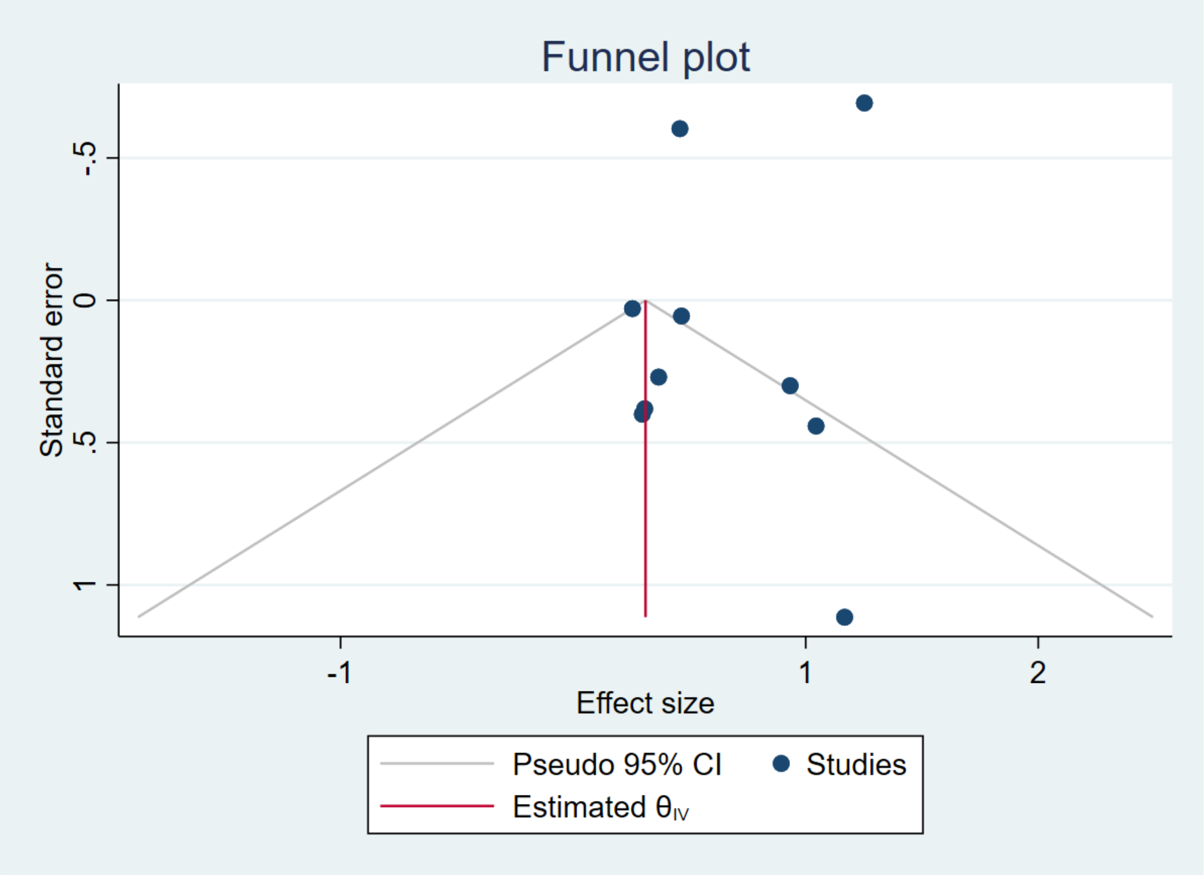

Source data are provided as a Source Data file.

Egger's test for small study effects on the outcome of pre-eclampsia

Number of studies = 34 Root MSE = 1.197

Std_Eff	Coefficient	Std. err.	t	P>|t|	[95% conf. interval]	
slope	0.8668596	0.1397917	6.20	0.000	0.5821132	1.151606
bias	0.0531521	0.3392992	0.16	0.877	-0.6379778	0.744282

Test of H0: no small-study effects P = 0.877

Supplementary Figure 6_a. Forest Plot of association of polycystic ovary syndrome with eclampsia

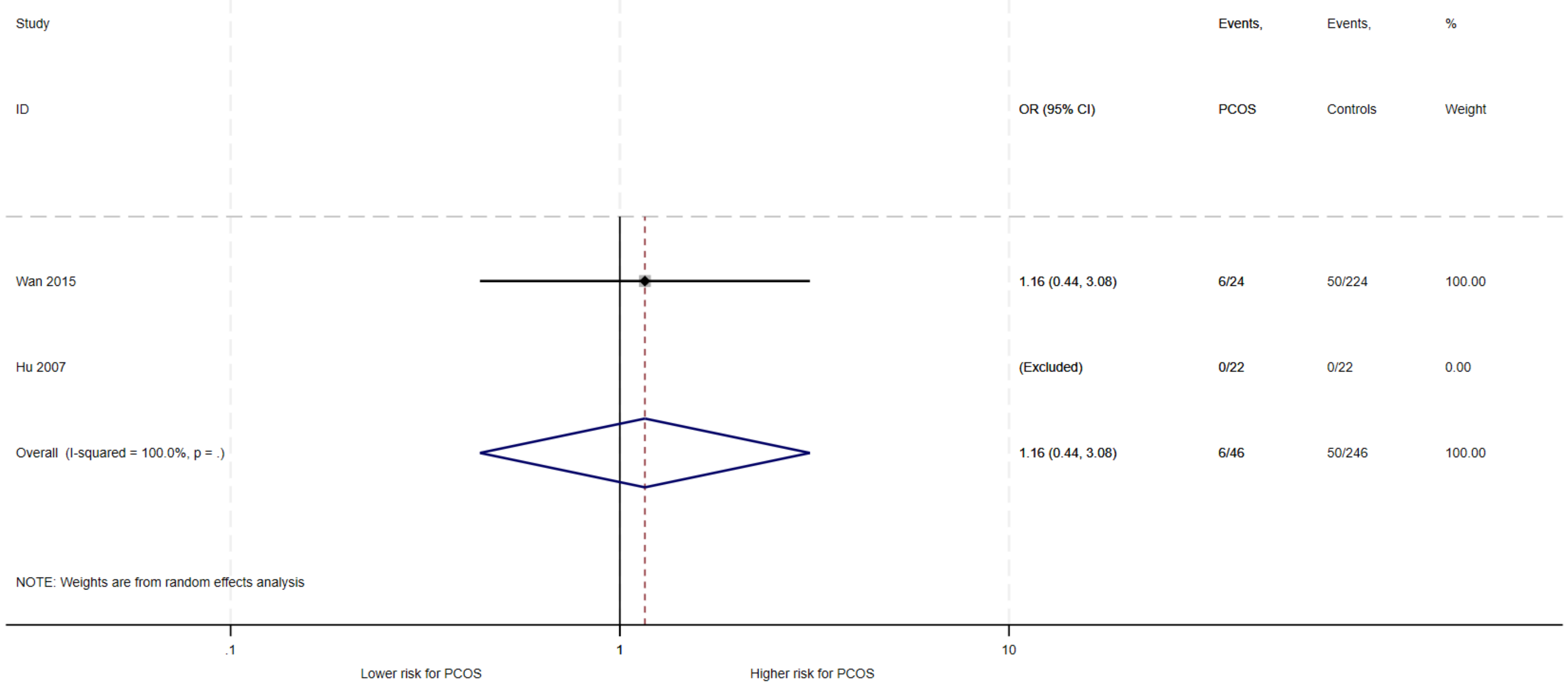

Supplementary Figure 6_b. Cumulative plot of association of polycystic ovary syndrome with eclampsia

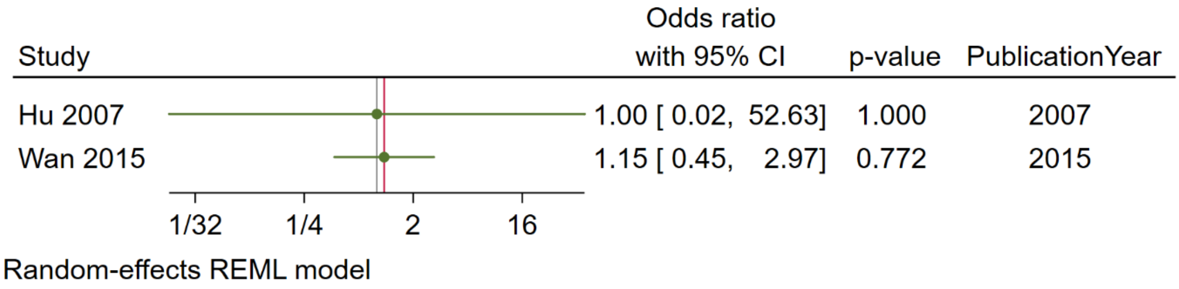

Source data are provided as a Source Data fil.

Supplementary Figure 7_a. Forest Plot of association of polycystic ovary syndrome with induction of labor

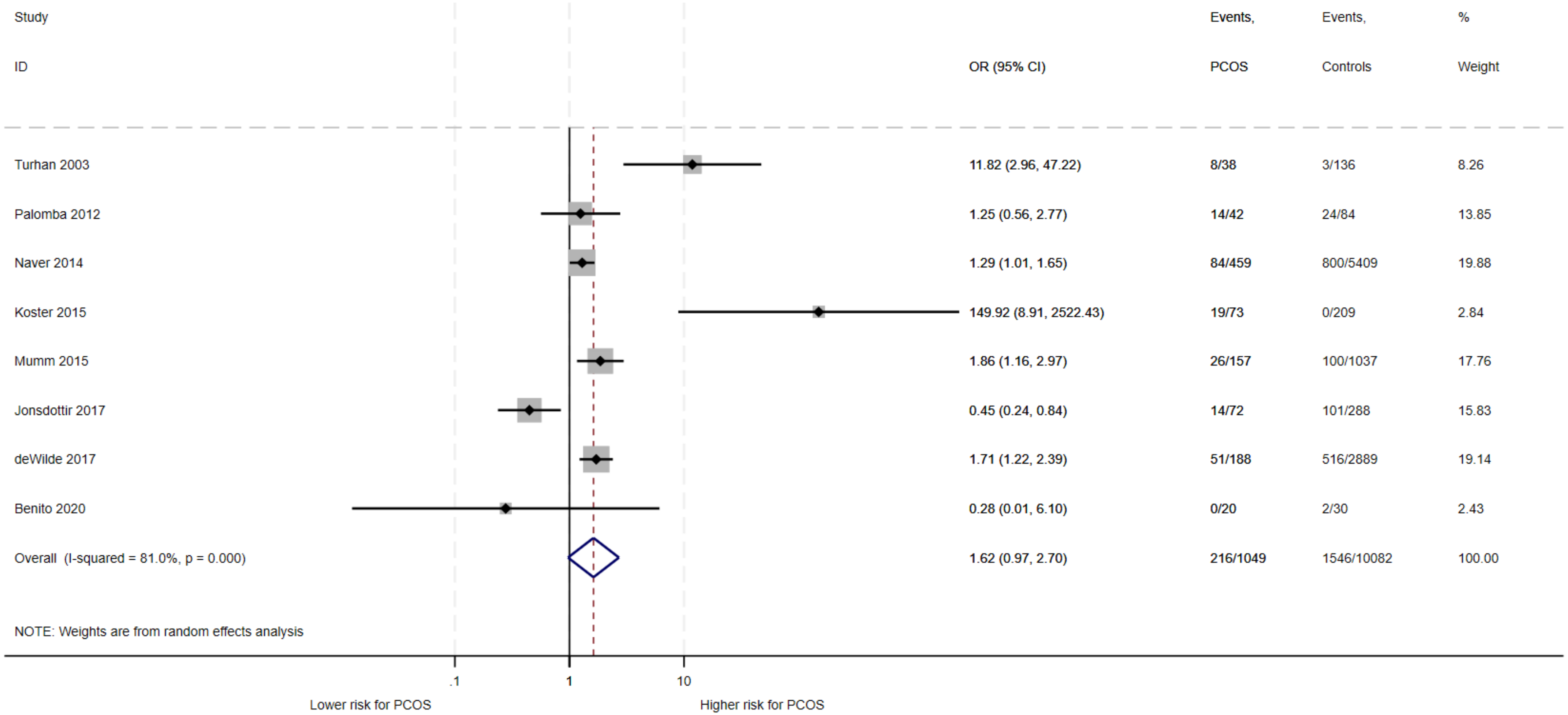

Supplementary Figure 7_b. Cumulative plot of association of polycystic ovary syndrome with induction of labor

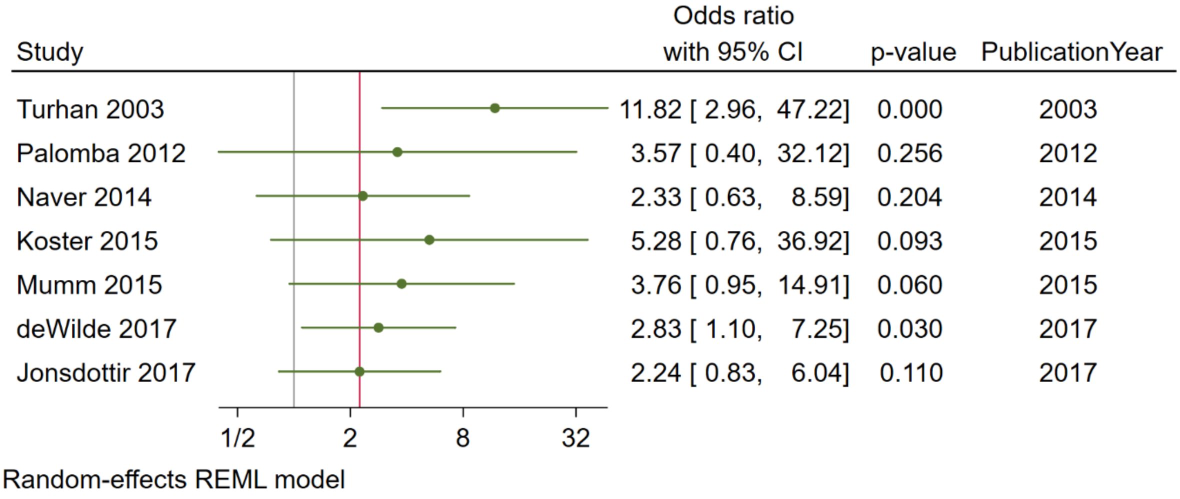

Supplementary Figure 7_c. Funnel plot assessing publication bias in studies on the association of polycystic ovary syndrome with induction of labor

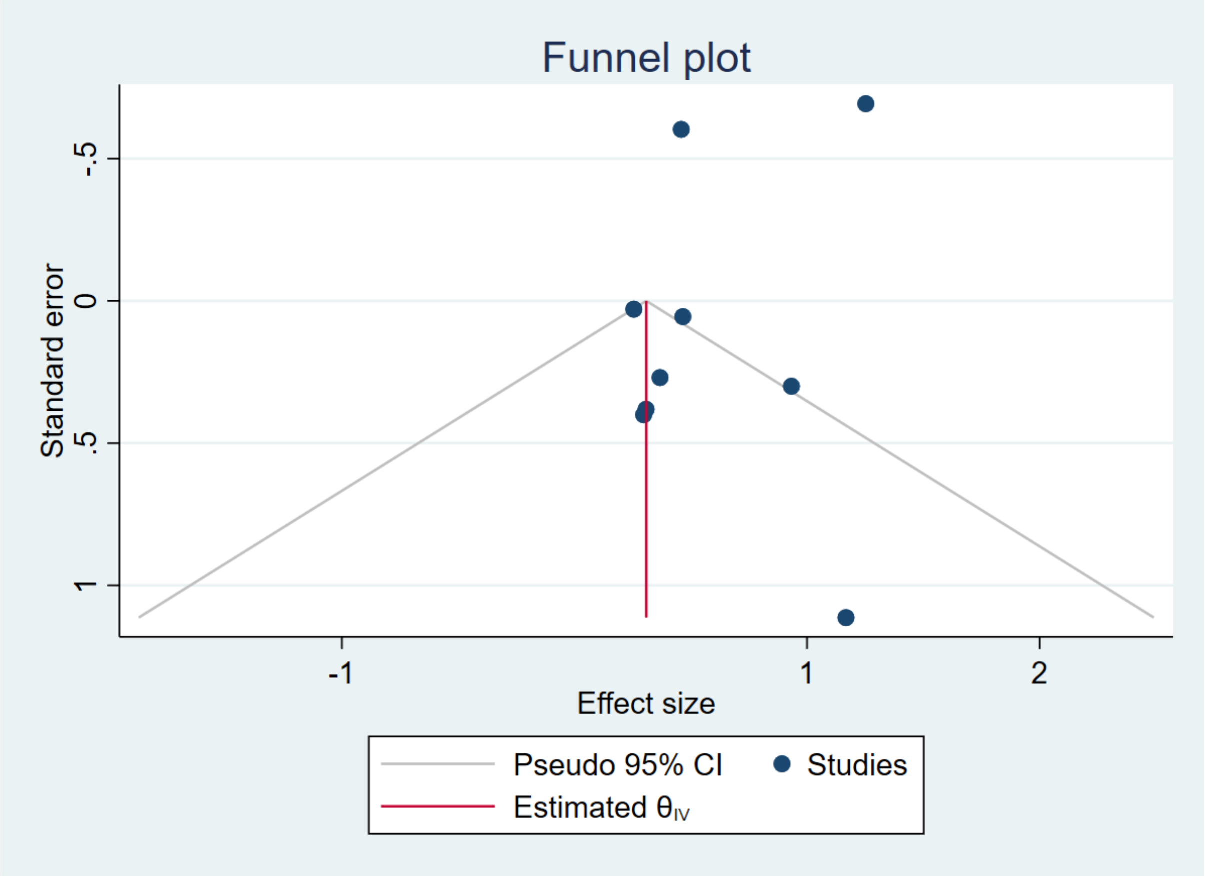

Source data are provided as a Source Data file

Supplementary Figure 8_a. Forest Plot of association of polycystic ovary syndrome with instrumental delivery

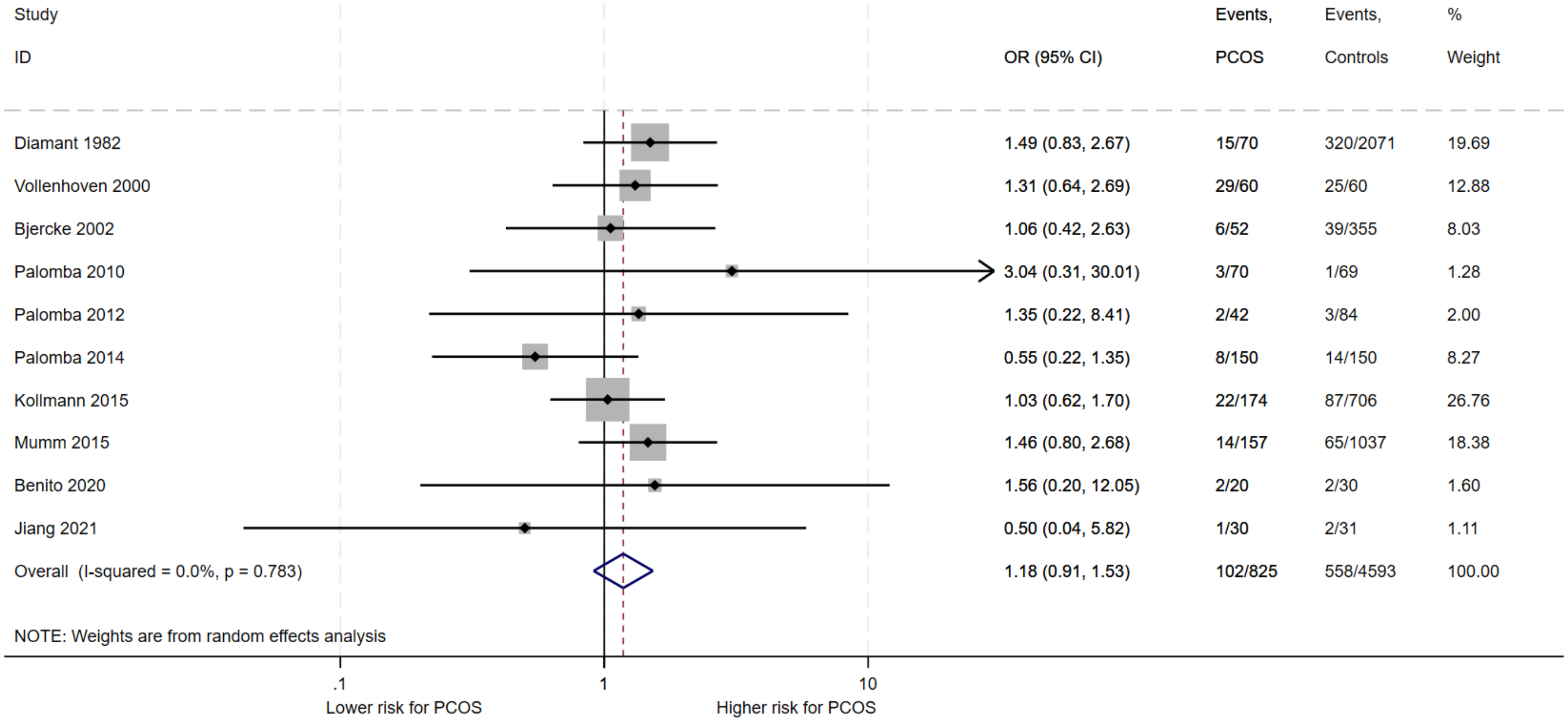

Supplementary Figure 8_b. Cumulative plot of association of polycystic ovary syndrome with instrumental delivery

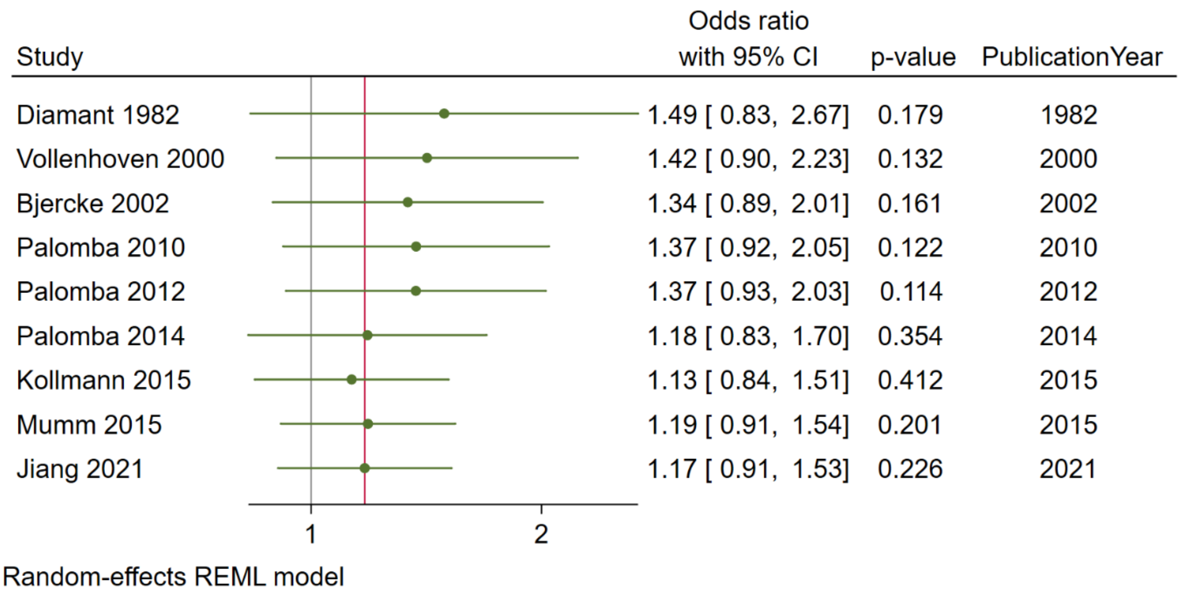

Supplementary Figure 8_c. Funnel plot assessing publication bias in studies on the association of polycystic ovary syndrome with instrumental delivery

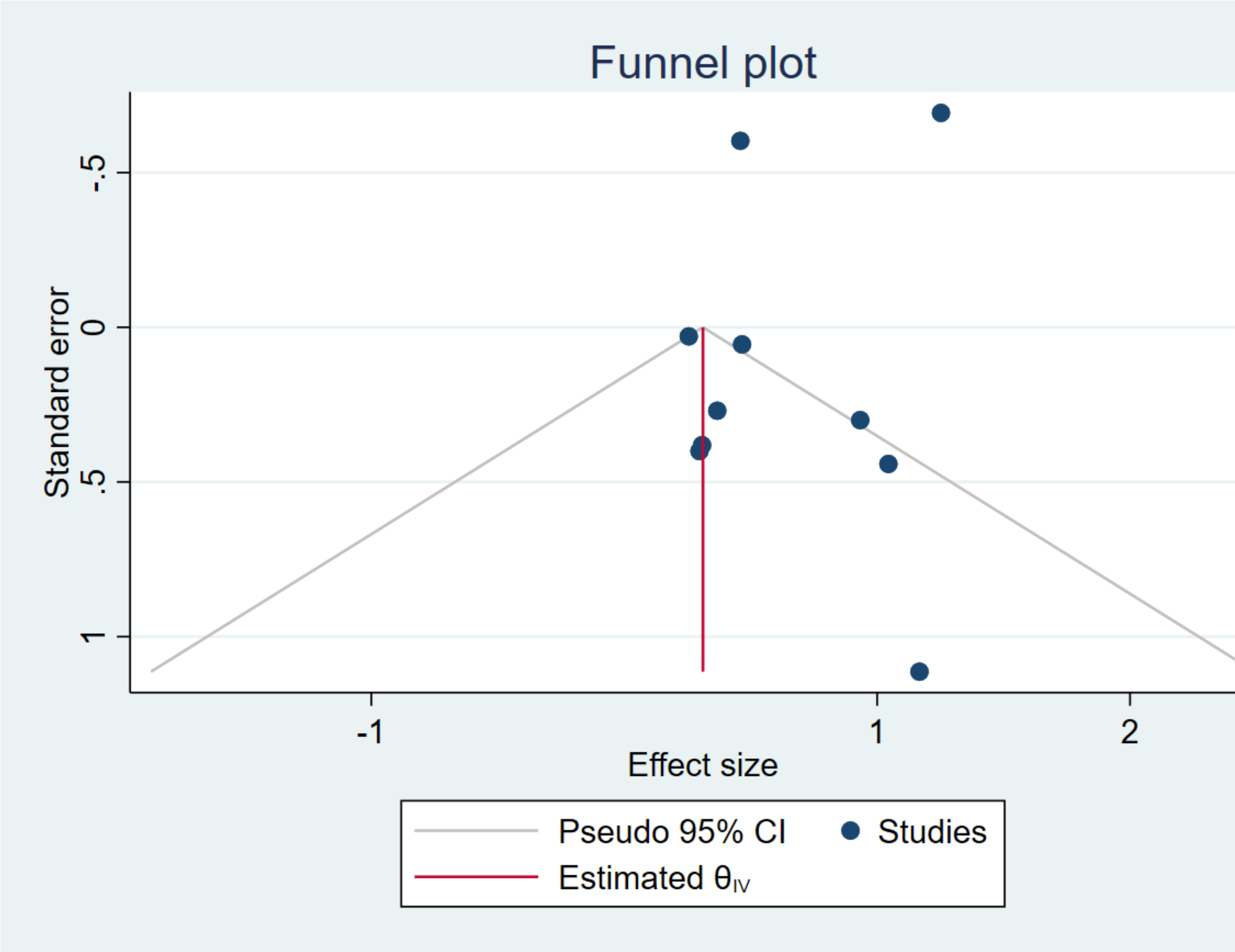

Source data are provided as a Source Data file.

Egger's test for small study effects on the outcome of instrumental delivery

Number of studies = 10 Root MSE = 0.8325

Std_Eff	Coefficient	Std. err.	t	P>|t|	[95% conf. interval]	
slope	0.1899516	0.2383662	0.80	0.449	-0.3597218	0.7396251
bias	-0.0654463	0.5708984	-0.11	0.912	-1.38194	1.251048

Test of H0: no small-study effects P = 0.912

Supplementary Figure 9_a. Forest Plot of association of polycystic ovary syndrome with cesarean section

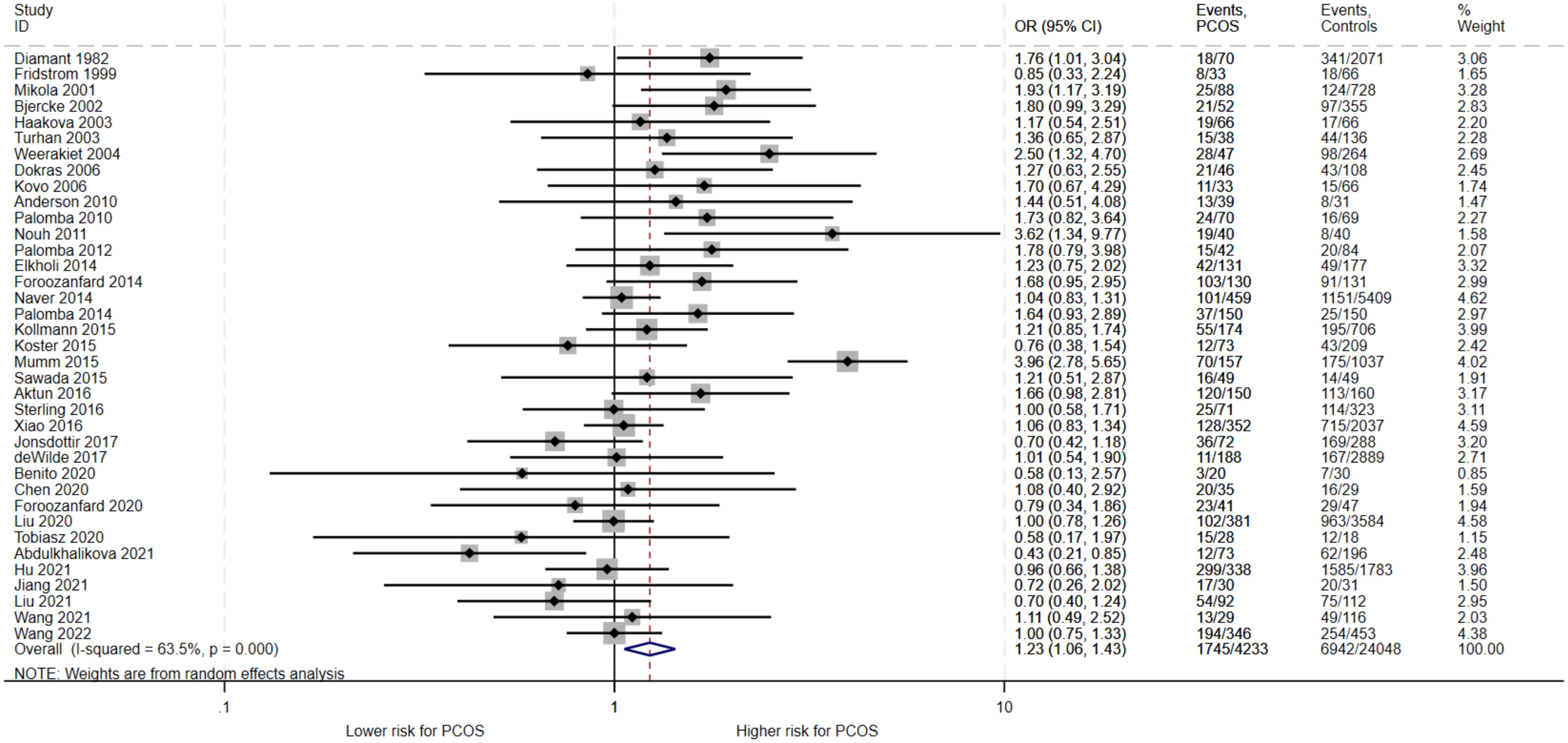

Supplementary Figure 9_b. Cumulative plot of association of polycystic ovary syndrome with cesarean section

Convergence not achieved during tau^2 estimation

Supplementary Figure 9_c. Funnel plot assessing publication bias in studies on the association of polycystic ovary syndrome with cesarean section

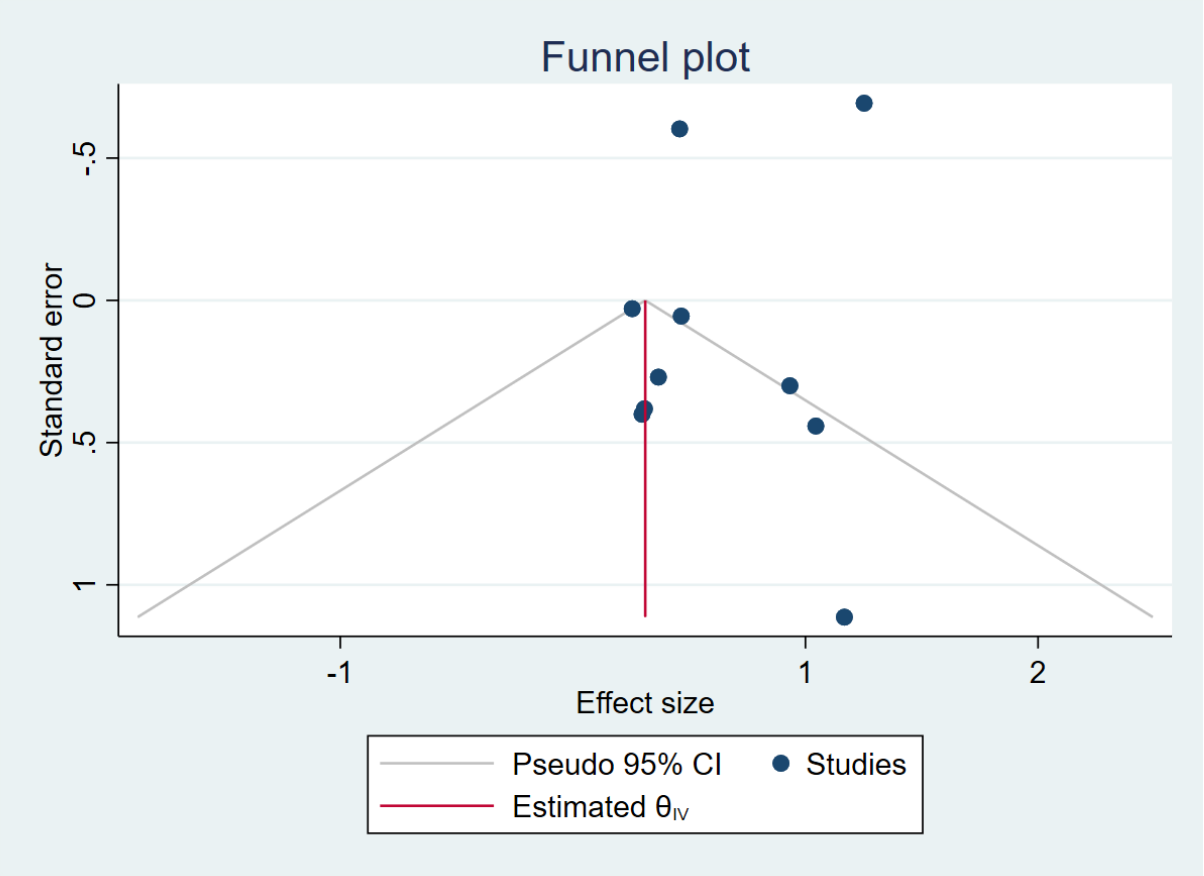

Source data are provided as a Source Data file.

Egger's test for small study effects on the outcome of cesarean section

Number of studies = 37 Root MSE = 1.673

Std_Eff	Coefficient	Std. err.	t	P>|t|	[95% conf. interval]	
slope	0.1212767	0.1488741	0.81	0.421	-0.1809538	0.4235072
bias	0.2696175	0.5954514	0.45	0.653	-0.939213	1.478448

Test of H0: no small-study effects P = 0.653

Supplementary Figure 10_a. Forest Plot of association of polycystic ovary syndrome with perinatal depression

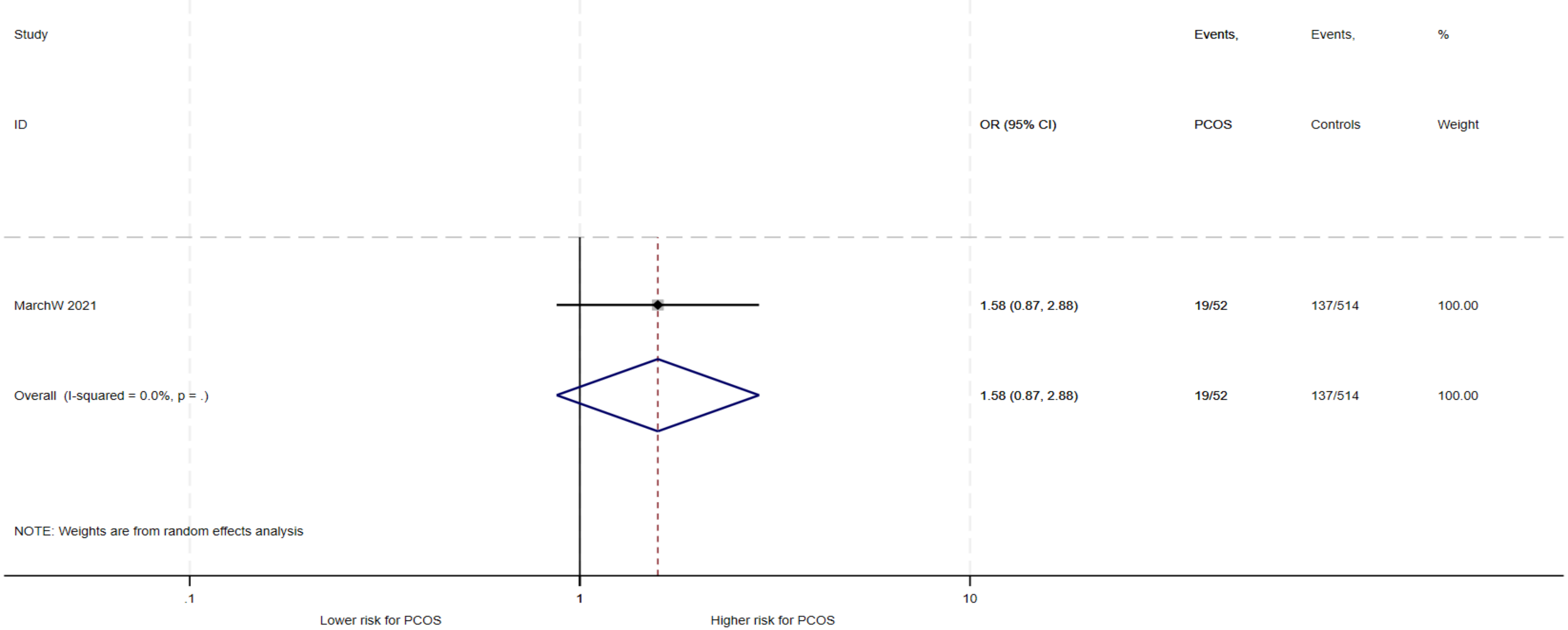

Source data are provided as a Source Data file.
